# Revisiting fecal metatranscriptomics analyses of macaques with idiopathic chronic diarrhoea with a focus on trichomonad parasites

**DOI:** 10.1017/S0031182022001688

**Published:** 2023-03

**Authors:** Nicholas P. Bailey, Robert P. Hirt

**Affiliations:** Biosciences Institute, Newcastle University, Catherine Cookson Building, Framlington Place, Newcastle-upon-Tyne, NE2 4HH, UK

**Keywords:** Macaque, metatranscriptomics, *Pentatrichomonas*, *Tetratrichomonas*, *Trichomitus*, Trichomonads

## Abstract

Trichomonads, anaerobic microbial eukaryotes members of the phylum Parabasalia, are common obligate extracellular symbionts that can lead to pathological or asymptomatic colonization of various mucosal surfaces in a wide range of animal hosts. Results from previous *in vitro* studies have suggested a number of intriguing mucosal colonization strategies by Trichomonads, notably highlighting the importance of interactions with bacteria. However, *in vivo* validation is currently lacking. A previous metatranscriptomics study into the cause of idiopathic chronic diarrhoea in macaques reported the presence of an unidentified protozoan parasite related to *Trichomonas vaginalis*. In this work, we performed a reanalysis of the published data in order to identify the parasite species present in the macaque gut. We also leveraged the information-rich metatranscriptomics data to investigate the parasite behaviour *in vivo*. Our results indicated the presence of at least 3 genera of Trichomonad parasite; *Tetratrichomonas*, *Pentatrichomonas* and *Trichomitus*, 2 of which had not been previously reported in the macaque gut. In addition, we identified common *in vivo* expression profiles shared amongst the Trichomonads. In agreement with previous findings for other Trichomonads, our results highlighted a relationship between Trichomonads and mucosal bacterial diversity which could be influential in health and disease.

## Graphical abstract

**Figure:**
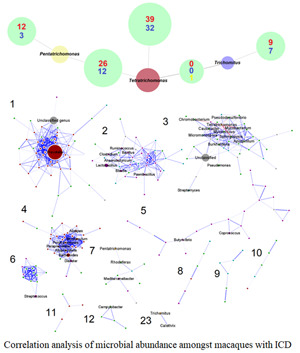

## Introduction

Trichomonads are a group of microbial eukaryotes within the phylum Parabasalia, almost all of which are known as obligate mucosal symbionts that colonize a wide range of mammals, birds and reptiles (Malik *et al*., 2011). Parasitic Trichomonads have no known free-living stages and are assumed to be transmitted almost exclusively by direct contact. The molecular basis of virulence, mucosal colonization (Sommer *et al*., 2005; de Miguel *et al*., 2010; Handrich *et al*., 2019; Martínez-Herrero *et al*., 2019) and metabolism (Matthews, 1986; Westrop *et al*., 2017) of Trichomonads has been the subject of extensive *in vitro* investigation. The vast majority of this work has focused on the human sexually transmitted parasite *Trichomonas vaginalis*. However, the importance of the proposed mechanisms during colonization of the complex mucosal environment *in vivo* is unclear. Validation of hypotheses in the natural setting is essential to avoid misinterpretation of results (Bello-Ortí *et al*., 2015; Marzano *et al*., 2017). The conservation of genes encoding virulence and mucosal colonization mechanisms across a wider range of Trichomonad species is also largely unknown, necessitating comparative studies.

There is extensive evidence for an interaction between Trichomonads and mucosal bacteria which is influential in health and disease. For example, *T. vaginalis* infection can induce dysbiotic changes in the urogenital tract (UGT) microbiota (Fichorova *et al*., 2013). Such results have been validated *in vivo* in several Trichomonad species and hosts (Wei *et al*., 2020; Bierlein *et al*., 2021). Notably, *Trichomonas gallinae* infection was correlated with changes in the microbiota at local and distant mucosal sites in pigeon squabs (Ji *et al*., 2020). However, previously, methods have been exclusively limited to 16S profiling, which provides no information on the potential mechanisms underlying parasite–bacteria interactions. *T. vaginalis* is a phagocytic predator of bacteria (Juliano *et al*., 1991; Rendon-Maldonado *et al*., 1998) and fungi (Pereira-Neves and Benchimol, 2007), with some evidence for selective preference of prey species (Juliano *et al*., 1991). In addition, *T. vaginalis* can form symbiotic associations with *Mycoplasma* spp. (Dessì *et al*., 2019). Functional work is required to determine the contribution of predation, symbiosis or other mechanisms to Trichomonad-induced *in vivo* microbiota changes.

A recent fecal metatranscriptomics investigation by Westreich *et al*. (2019) into the cause of idiopathic chronic diarrhoea (ICD) in laboratory macaques revealed the presence of GIT-localized protozoa. The authors stated ‘Protozoans with the most abundant transcripts in the faecal samples from the macaques were *Blastocystis* sp. and *Trichomonas vaginalis*’; however, also qualified ‘*T. vaginalis* was the only species in the reference data set representative of the *Trichomonas* genus, so it is possible that the particular species with increased gene expressed in macaques with ICD was not *T. vaginalis*’. Trichomonads do exhibit mucosal and host plasticity (Maritz *et al*., 2014). For example, *T. vaginalis* has been detected in the oral cavity (Costello *et al*., 2017) and respiratory tract (Duboucher *et al*., 2003). In addition, *T. vaginalis* is thought to originate from zoonosis of an avian oral parasite (Maritz *et al*., 2014). However, *T. vaginalis* is essentially a human UGT parasite. We suggest that misidentification as ‘*T. vaginalis*’ due to sequence database incompleteness (Watts *et al*., 2019) is very likely, as there are no reports of *T. vaginalis* in non-human animals or the GIT. Thus, further investigation into the parasite identity is warranted. Studies conducted on wild macaques did not report the presence of intestinal Trichomonads (Adhikari and Dhakal, 2018), although infection with *Pentatrichomonas hominis* has been reported in immunocompromised laboratory macaques (Zaragoza *et al*., 2011) and those suffering from ICD (Laing *et al*., 2018). Thus, *P. hominis* is a likely candidate for the parasite infecting the macaques with ICD.

In this work, we utilized the existing fecal metatranscriptomics data from macaques (Westreich *et al*., 2019) to identify the Trichomonads present, and to investigate their *in vivo* gene expression. Our results also suggested relationships between Trichomonads and the mucosal microbiota *in vivo*, with potential implications for the aetiology of ICD.

## Materials and methods

### Macaque fecal metatranscriptomics data analysis

Full details for experimental methodology used to generate the previously published macaque fecal metatranscriptomics data are available from Westreich *et al*. (2019). Briefly fecal samples were collected from 12 macaques suffering from ICD and 12 healthy control animals. For 30 days prior to sample collection animals were housed indoors in pairs, separating macaques with ICD and healthy controls. For stool collection, cage pans were placed under the cages of individually housed animals overnight and collected in the morning. Stool samples were used as a proxy for the intestinal mucosal environment. Total RNA was extracted from stool samples and used for cDNA library preparation.

The workflow used to assess macaque fecal metatranscriptomics data is shown in Fig. S1. The metatranscriptomics dataset for macaques was obtained from the NCBI SRA database (Leinonen *et al*., 2011) under accessions SRX3517701-SRX3517724 (Westreich *et al*., 2019). There were approximately 95 million paired end reads per animal. The average Phred quality score for all reads did not fall below 30, as assessed using FastQC version 0.11.9 (Andrews, 2018). For quantitative analysis of taxonomic abundance and functional expression, reads derived from rRNA were filtered by alignment to a prokaryotic and eukaryotic rRNA database using SortMeRNA version 4.2.0 (Kopylova *et al*., 2012). If both reads in a pair aligned, the pair was excluded. Kraken2 version 2.0.8-beta (Wood *et al*., 2019) with default parameters was used to taxonomically classify reads. The Kraken2 reference database was enriched by including *de novo* assembled contigs derived from *in vitro* RNA-Seq data for *P. hominis* strain PhGII, *Tetratrichomonas gallinarum* strain M3, and *Trichomitus batrachorum* strain BUB, kindly provided by Sriram Garg and Sven Gould (Handrich *et al*., 2019) (Heinrich Heine University, Düsseldorf) to improve the Trichomonad sequence diversity. The NCBI Taxonomy Toolkit version 0.5.0 (Shen and Xiong, 2019) was used to manipulate taxonomy IDs generated by Kraken2.

A *de novo* assembly was generated from the data using metaSPAdes version 3.13.0 (Nurk *et al*., 2017). To assess the accuracy of the assembly, reads were aligned to assembled contigs using STAR version 2.7.3a (Dobin *et al*., 2013). Samtools version 0.1.20 (Li *et al*., 2009) was used to manipulate alignment files. Overview for the dataset and assembly are presented in Table S1. *De novo* assembled contigs derived from combined Parabasalia reads from all samples to maximize coverage (available in Data files S1, selected genes, and S2, all genes) were used to examine Parabasalia gene expression. For transcript annotation, assembled parasite contigs were aligned to the *T. vaginalis* G3 annotated proteins (Carlton *et al*., 2007) using BLASTx version 2.9.0+ (Altschul *et al*., 1990). A single top hit for each contig was selected after sorting by, respectively and in priority order, E value and percentage identity, and excluding hits with an E value greater than 1 × 10^−10^ or a query coverage of less than 70%. Annotated contigs are presented in Table S2 (specific genes used as phylogenetic markers) and Table S3 (all genes).

### Phylogenetic analysis

To sequence type parasites, BLASTn version 2.9.0+ (Altschul *et al*., 1990) was used to identify *de novo* assembled contigs homologous to Parabasalia genes of interest, with E value, percentage identity and query coverage cut-off values of 1 × 10^−10^, 88% and 90%, respectively. To broaden the taxonomic sampling for genes of interest, additional homologues were identified by consulting the literature and through the use of online BLAST (Altschul *et al*., 1990) searches against the NCBI non-redundant protein or nucleotide databases (O'Leary *et al*., 2016). Alignments were generated using Clustal omega version 1.7 (Sievers *et al*., 2011) and visually inspected in Seaview version 5.0.4 (Gouy *et al*., 2010). To improve phylogenetic resolution of parasite sequencing typing, DNA alignments for protein-coding genes were generated. Protein sequences were aligned using Clustal omega version 1.7 (Sievers *et al.*, 2011), and corresponding codon alignments were derived using pal2nal version 14 (Suyama *et al*., 2006). Poorly aligned sequences were removed, and alignments were trimmed to remove excessive gaps (sites containing a gap for greater than 90–95% of sequences) using TrimAl version 1.2 (Capella-Gutiérrez *et al*., 2009). Alignments are available in supplementary data files S3–S8. IQ-tree version 1.6.1 (Nguyen *et al*., 2015) was used to generate maximum likelihood phylogenies, using automatic model selection. Support for tree topology was assessed by computing 1000 bootstrap replicates. iTol (Letunic and Bork, 2019) was used to generate annotated figures from the phylogenies.

### Microbial diversity and expression analysis

Microbial diversity analysis was performed using the R packages PhyloSeq version 1.34.0 (McMurdie and Holmes, 2013) and Microbiome version 1.12.0 (Lahti and Shetty, 2017), excluding reads assigned within equivalent or child taxa to animals, viruses or Parabasalia. ANCOM-BC version 1.0.5 (Lin and Peddada, 2020) was used for differential abundance analysis, excluding reads assigned within equivalent or child taxa to animals or plants. SparCC (Friedman and Alm, 2012) was used for microbial correlation analysis, including only bacterial and parabasalid genera representing at least 0.005% of the sequencing library in at least 1 sample. Boostrapped samples (100 replicates) of microbial abundance were used to calculate 2-sided pseudo-*P* values. Microbial correlation networks were derived from the sparCC results using the R package igraph version 1.2.11 (Csardi and Nepusz, 2006), with edges linking genera sharing a correlation coefficient greater than 0.8 and pseudo *P* value lower than 0.05. Networks were split into modular components by the Louvain method (Blondel *et al*., 2008) and Cytoscape version 3.6.1 (Shannon *et al*., 2003) was used to generate figures and calculate network summary statistics. For functional analysis of microbial transcription, reads assigned within equivalent or child taxa to animals, plants, Parabasalia or viruses were excluded. HUMAnN version 2.8.2 (Franzosa *et al*., 2018) was used to functionally classify reads at the gene family level by translated alignment to UniRef90 protein families (Suzek *et al*., 2015). Gene family abundance values were normalized to library size (in counts per million; CPM) prior to assignment to MetaCyc pathways (Caspi *et al*., 2016) to calculate pathway abundance using HUMAnN version 3.0.0 (Beghini *et al*., 2021). Pathway abundance values were log_2_ transformed, with an added pseudocount of 0.01, before differential abundance test by the limma-trend method (Law *et al*., 2014).

## Results

### Identity of trichomonads colonizing the macaque gut

We performed an analysis on the unidentified Trichomonads which were reported in published fecal metatranscriptomics data from rhesus macaques with ICD (Westreich *et al*., 2019). Metatranscriptomes were available for 12 macaques with ICD (Macaques 1–12) and 12 healthy control animals (Macaques 13–24).

We aimed to investigate the identity of Trichomonad parasites reportedly present in this dataset (Westreich *et al*., 2019). To generate sequences for molecular typing, we generated a *de novo* assembly of the metatranscriptome. Contig length statistics suggested an overall low degree of assembly, with a mode contig length of 161 bp across all samples, and N50 values ranging from 544 to 1021 bp. Alignment of reads to the assembly indicated no major compositional biases (Fig. S2). Summary statistics for the dataset and assembly are presented in Table S1. The *de novo* assembly is available in Data file S2.

**Fig. 1.**
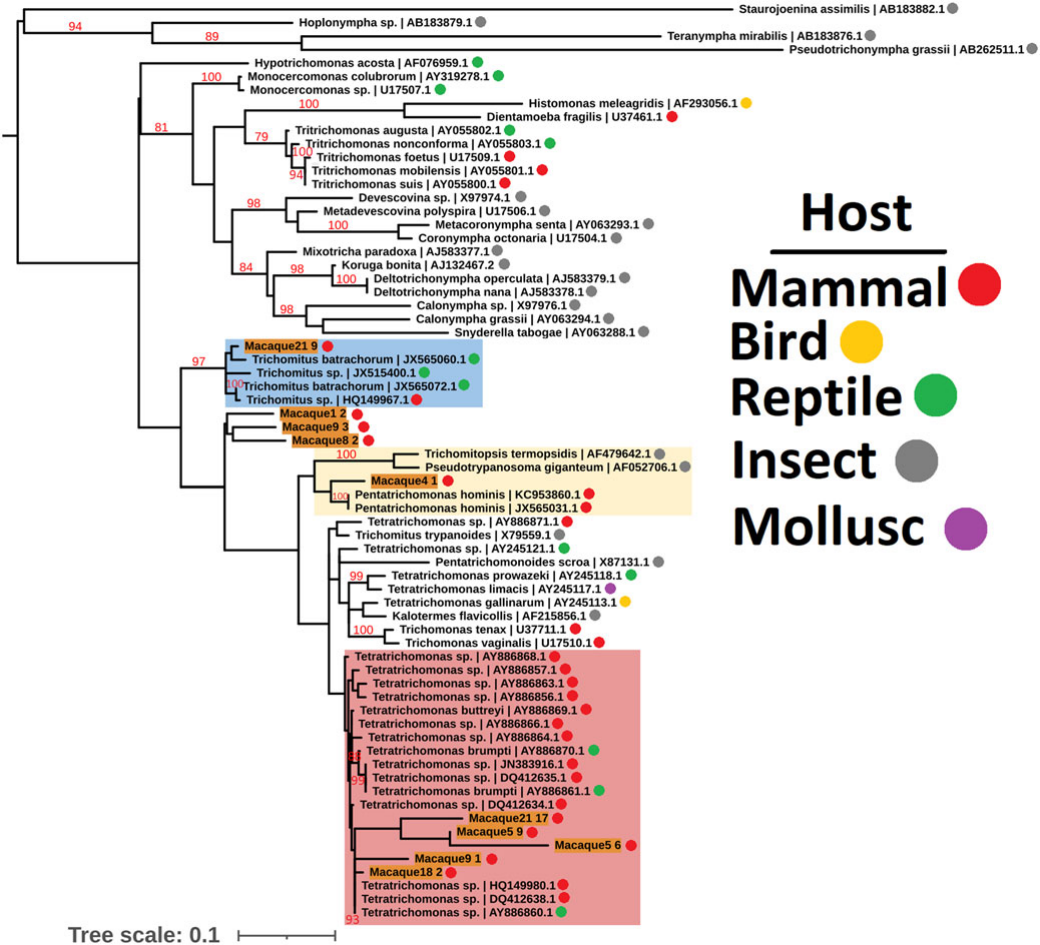
Unrooted maximum likelihood phylogeny (GTR model with empirical base frequencies, invariable sites and the discrete gamma model) of Parabasalia-like 18S rRNA sequences from the macaque fecal metatranscriptome, alongside a range of Parabasalia species. Bootstrap values (1000 replicates) greater than 75% are shown on branches in red. Units for tree scale are inferred substitutions per base pair. Macaque-derived sequences (highlighted in orange) are named sequentially according to the animal from which they originated, e.g. Macaque1:1, Macaque1:2. Major lineages of macaque-derived *Tetratrichomonas*-like, *Pentatrichomonas*-like and *Trichomitus*-like sequences are highlighted in red, yellow and blue, respectively. Where available, Genbank accessions (Benson *et al*., 2015) are shown at the ends of tip labels.

**Fig. 2.**
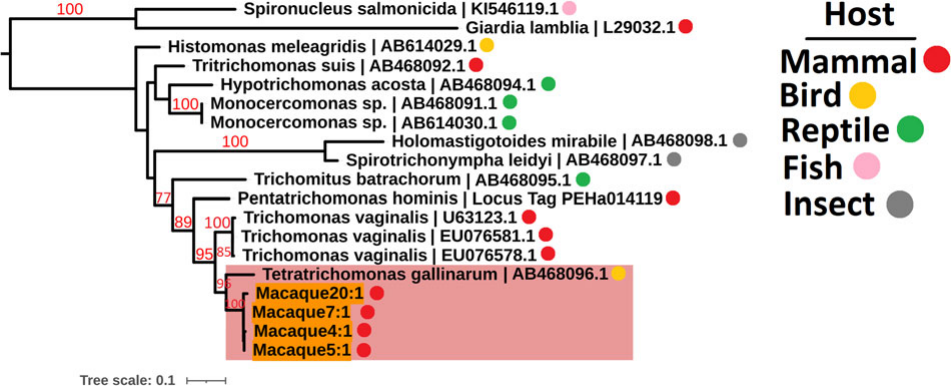
Maximum likelihood phylogeny (TIM2e model with equal base frequencies and the discrete gamma model) of Parabasalia-like actin sequences from the macaque fecal metatranscriptome alongside a range of Parabasalia species. Phylogeny is rooted using sequences from *Giardia lamblia* (accession L29032.1) and *Spironucleus salmonicida* (accession KI546119.1) as an outgroup (not shown). Bootstrap values (1000 replicates) greater than 75% are shown on branches in red. Units for tree scale are inferred substitutions per base pair. Macaque-derived sequences (highlighted in orange) are named sequentially according to the animal from which they originated, e.g. Macaque1:1, Macaque1:2. The major *Tetratrichomonas*-like lineage of macaque-derived sequences is highlighted in red. Coloured dots indicate animal host taxa. Where available, Genbank accessions (Benson *et al*., 2015) are shown at the ends of tip labels.

**Fig. 3.**
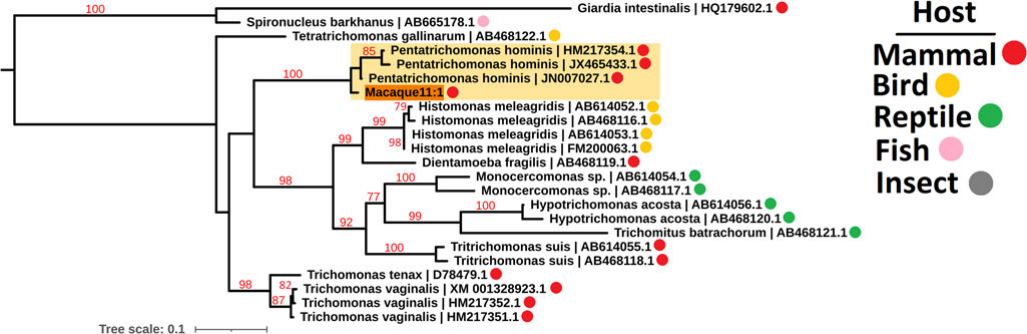
Maximum likelihood phylogeny (TIM2 model allowing unequal base frequencies, with empirical base frequencies, invariable sites and the discrete gamma model) of Parabasalia-like EF-1*α* sequences from the macaque fecal metatranscriptome, alongside a range of Parabasalia species. Phylogeny is rooted using sequences from *Giardia intestinalis* (accession HQ179602.1) and *Spironucleus barkhanus* (accession AB665178.1) as an outgroup (not shown). Bootstrap values (1000 replicates) greater than 75% are shown on branches in red. Units for tree scale are inferred substitutions per base pair. Macaque-derived sequences (highlighted in orange) are named sequentially according to the animal from which they originated, e.g. Macaque1:1, Macaque1:2. The major *Pentatrichomonas*-like lineage of macaque-derived sequences is highlighted in yellow. Coloured dots indicate animal host taxa. Where available, Genbank accessions (Benson *et al*., 2015) are shown at the ends of tip labels.

Due to the low sequence coverage and high complexity of parasite sequences, we utilized the 18S rRNA, actin and elongation factor 1 alpha (EF-1*α*) loci to identify the Parabasalia colonizing the macaque gut. Amongst all the samples, we assembled 58, 10 and 11 18S rRNA, actin and EF-1*α* sequences, respectively, which shared greater than 88% sequence identity with reference Parabasalia sequences for at least 90% of their length (Table S2). We assessed the diversity of sequences present by maximum likelihood analysis and identified 10 well-supported clades for the 18S rRNA locus (Fig. S3), 4 for the actin locus (Fig. S4), and 1 for the EF-1*α* locus (Fig. S5). We generated phylogenies using representative sequences from each clade alongside a range of Parabasalia reference sequences in order to refine the identity of the parasite sequences. Analysis of a single representative sequence from each of the 18S rRNA sequence groups revealed at least 3 major lineages, related to *Tetratrichomonas*, *Pentatrichomonas* and *Trichomitus* spp., with strong bootstrap support only present for the latter (99%; Fig. 1). In contrast, there was strong bootstrap support (99%) for grouping of all identified actin sequences with *Tetratrichomonas gallinarum* (Fig. 2), and all identified EF-1*α* sequences with *P. hominis* (100%; Fig. 3). Integrating these analyses, we inferred that there are likely to be 3 Parabasalia lineages, related to *Trichomitus*, *Tetratrichomonas* and *Pentatrichomonas*, present amongst the macaque fecal samples.

**Fig. 4.**
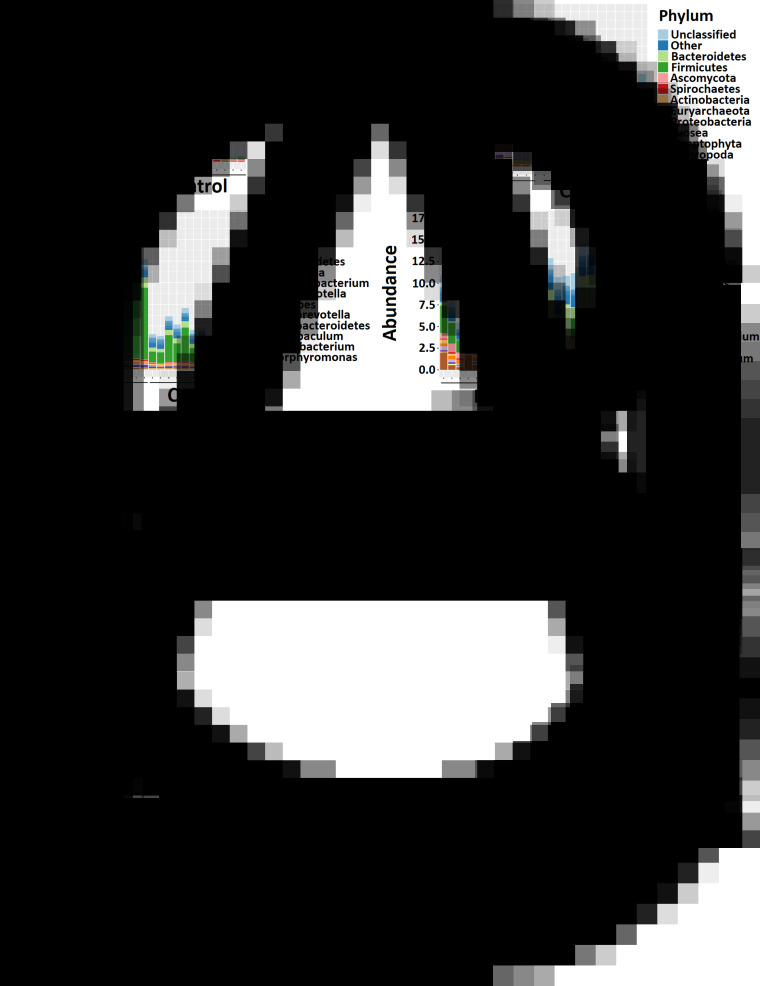
Summary of microbial abundances amongst control macaques and this with idiopathic chronic diarrhoea. (A) Parabasalia genera of interest, (B) Phyla excluding the host or Parabasalia (C) Bacteroidetes genera and (D) Firmicutes genera. (B-D) show the abundance of the top 10 most abundant taxa (sum across all samples). Abundances are presented as a percentage of the total sequence library size. The ‘other’ category groups the rest of taxa not shown, and lines separate the subdivisions within these bars. The ‘unclassified’ category represents sequence reads which have been assigned to the relevant taxon of interest for the plot, but not to any specific phylum or genus. Samples are ordered 1–24 from left to right.

To taxonomically assign the metatranscriptome reads, we included *de novo* assembled contigs derived from *in vitro* RNA-Seq analysis of *P. hominis*, *Tetratrichomonas gallinarum* and *Trichomitus batrachorum* (Handrich *et al*., 2019) in the reference database to improve assignment for sequences derived from the putative parasite genera of interest. In agreement with the phylogenetic results, *Trichomitus, Pentatrichomonas* and *Tetratrichomonas* were the 3 most abundant (mean across all samples) parabasalid genera which were identified (Fig. 4A). According to read assignment, *Trichomitus* was the most abundant individual genus of interest (mean abundance 0.096%), followed by *Pentatrichomonas* (mean abundance 0.025%) and *Tetratrichomonas* (mean abundance 0.020%). A substantial number of sequences (mean abundance 0.093%) were identified as parabasalid in origin but could not be assigned to a particular genus. Unidentified parabasalid reads appeared more abundant among animals in which *Tetratrichomonas*, *Pentatrichomonas* or *Trichomitus* classified reads were abundant, likely suggesting that they originated from 1 or more of these genera.

In addition, a notable fraction of reads were classified as *Trichomonas* (mean abundance 0.019%). This most likely reflects the greater representation of *Trichomonas* whole genome sequences available in the reference database, including *T. vaginalis* and *T. gallinae*, whereas the genera of interest were only represented by *in vitro* RNA-Seq data, which is likely to have an incomplete gene content. However, while it cannot be ruled out that *Trichomonas* spp. were present amongst the samples, we have focused our analysis on the most likely genera based on the phylogenetic results. Only 2 control macaques showed a total abundance of Parabasalia greater than 0.125%, limiting the statistical power for tests correlating variables with Parabasalia abundance amongst the control animals.

### Trichomonad gene expression

We focused on the most abundant putative Parabasalia-derived contigs to explore the most biologically important functions, which are summarized in Table 1. Potential energy-generation pathways included glycolysis, hydrogenosome metabolism, catabolism of GlcNAc, GalNAc, galactose and glucosamine and amino acid catabolism, including the arginine dihydrolase (ADH) pathway. The presence of a putative xanthine dehydrogenase could also indicate catabolism of nucleotides as an additional nutrient source (Wang *et al*., 2016). Synthesis of glucose and storage as glycogen was suggested by gluconeogenesis and glycogen processing enzymes.

**Table 1.** Summary of selected Parabasalia-like contigs of interest derived from the macaque fecal metatranscriptome

Contig name	Annotation	Pathway/function	*T. vaginalis* hit % identity (protein level)	Mean abundance (RPM)	Rank (ranked by abundance of all Parabasalia-like contigs
NODE_1480	Glutamate dehydrogenase	Amino acid catabolism	56.1	6031	1
NODE_1122	Phosphoenol pyruvate carboxykinase	Gluconeogenesis	55.9	4187	3
NODE_1126	Starch branching enzyme	Starch synthesis	48.0	3211	4
NODE_791	Fructose-1,6-bisphosphate aldolase	Glycolysis	65.7	3006	6
NODE_638^a^	Glyceradehyde-3-phosphate dehydrogenase		85.3	2791	8
NODE_1712	Phosphoglucomutase/phosphomannomutase		42.4	2136	12
NODE_24	Pyruvate:ferredoxin oxidoreductase BII	Hydrogenosome metabolism	74.3	875	45
NODE_952	Ornithine carbamoyltransferase family protein	Arginine dihydrolase pathway	89.8	758	53
NODE_539	Malic enzyme	Hydrogenosome metabolism	83.6	289	200
NODE_134	Aldehyde oxidase and xanthine dehydrogenase	Purine catabolism	82.8	281	207
NODE_2461	Glucosamine-6-phosphate isomerase family protein	GlcNAc catabolism	49.8	265	222
NODE_2958	Transketolase family protein	Pentose phosphate pathway	45.9	242	239
NODE_418	Coronin	Phagocytosis	75.9	226	252
NODE_1833^a^	Lysozyme	Bacterial cell wall degradation	70.6	134	434
NODE_2008^a^	Lysozyme		64.8	126	462
NODE_589	Galactokinase family protein	Galactose metabolism	65.2	71	800
NODE_864	Succinyl-CoA ligase beta-chain	Hydrogenosome metabolism	78.1	60	946

a Annotation for the top *T. vaginalis* hit was uninformative, and so a lower hit within the top 10 hits was selected.

A number of Parabasalia contigs were annotated with putative lysozyme activity, thus potentially targeting the bacterial microbiota. A maximum likelihood phylogeny was generated to investigate the possibility that the contigs were bacterial in origin (Fig. S6). Results suggested that the lysozyme-like contig NODE_2008 originated from *Trichomitus* with strong support, and an additional contig NODE_1833 may have originated from *Pentatrichomonas* or *Tetratrichomonas*, although this was poorly supported. In addition, we identified a contig with high similarity to *T. vaginalis* coronin, an actin-binding protein implicated in phagocytosis (Bricheux *et al*., 2000), thus consistent with parasite phagocytosis targeting microbial or host cells.

Numerous contigs showed strong similarity to *T. vaginalis* genes previously implicated in pathobiology. Of particular interest for parasite adhesion to host or microbial cells (Handrich *et al*., 2019), we detected expression of 762 contigs with substantial sequence similarity to *T. vaginalis* BspA proteins. As the BspA family represent strong candidate LGTs of prokaryotic origin (Handrich *et al*., 2019), it is likely that this list could include bacterial contigs, due to high similarity between the Trichomonad and bacterial sequences. The list included 70 contigs with greater than 60% sequence identity with the nearest *T. vaginalis* homologue (by BLASTx). Cysteine peptidases are also implicated in *Trichomonas* pathobiology (Sommer *et al*., 2005) and 93 contigs were detected which shared high similarity with *T. vaginalis* Clan CA, family C1, cathepsin L-like cysteine peptidases. Of particular interest, 17 contigs were close homologues of TvCP39 (locus tag TVAG_298080; mean percentage identity 69%), a secreted cysteine peptidase demonstrated to induce host cell apoptosis (Arroyo *et al*., 2015).

### Trichomonad interactions with the microbiota

To further investigate potential interactions between parabasalid parasites and the microbiota, we examined the taxonomic composition of the samples (Fig. 4). We reproduced a microbiota profile which was in agreement with the previous report (Westreich *et al*., 2019); Bacteroidetes and Firmicutes were the most abundant phyla (Fig. 4B), the former largely dominated by the genus *Prevotella* (Fig. 4C) and the latter composed of a diverse range of genera (Fig. 4D). A large proportion of sequences could not be taxonomically classified at the phylum level (mean 66.7% of reads across all samples).

**Fig. 5.**
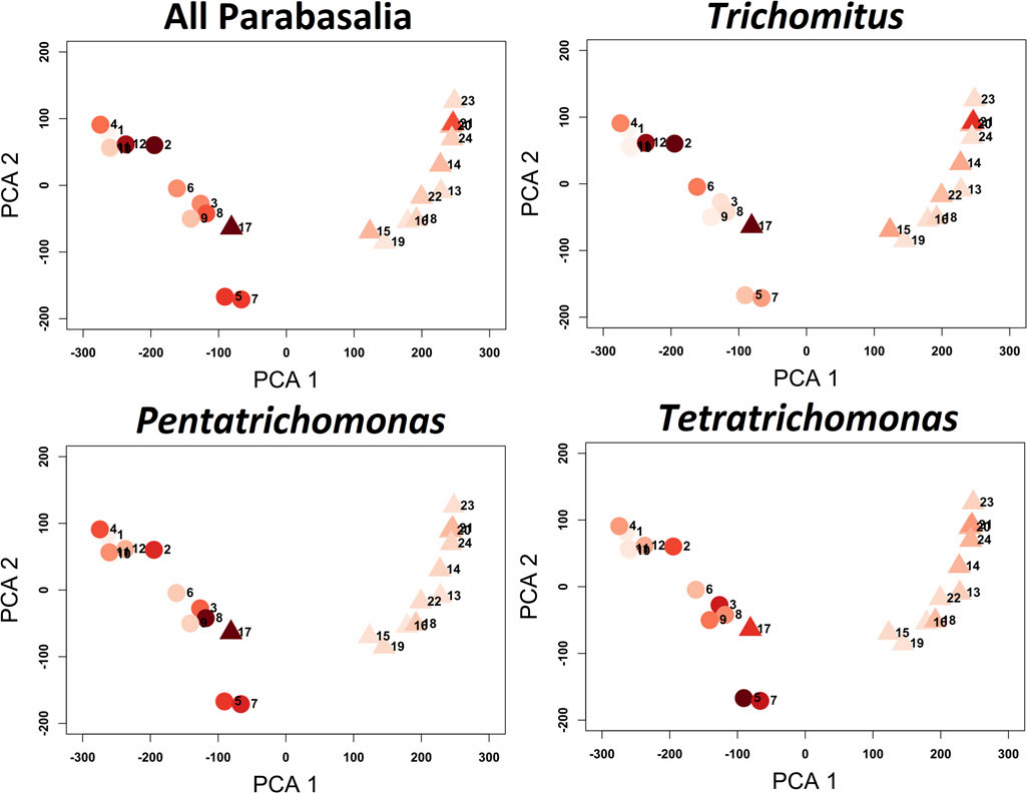
Principal component analysis (PCA) plot for Aitchison distance based on non-parabaslid microbial abundances amongst macaque fecal samples. Points are shaded according to the centred log ratio normalized abundance values for all Parabasalia, *Trichomitus*, *Pentatrichomonas* and *Tetratrichomonas*, with darker shades indicating greater abundance. Triangle and circular points indicate healthy and diseased animals, respectively.

Principal component analysis (PCA) based on the microbial profile showed clear separation between the healthy and ICD groups. The macaques with ICD appeared to resolve into 3 subgroups, potentially indicating distinct microbial communities (Fig. 5). An obvious association between PCA-based clustering and abundance of the parabasalid genera of interest was not clear. However, we tentatively suggest a loose clustering of diseased animals somewhat consistent with *Tetratrichomonas* abundance (Fig. 5B–D). Of particular interest, a single healthy control animal, macaque 17, clustered amongst the diseased animals. Macaque 17 showed the greatest abundance of total Parabasalia, *Trichomitus* and *Pentatrichomonas* of all macaques, and the greatest *Tetratrichomonas* abundance amongst the macaques with ICD.

**Fig. 6.**
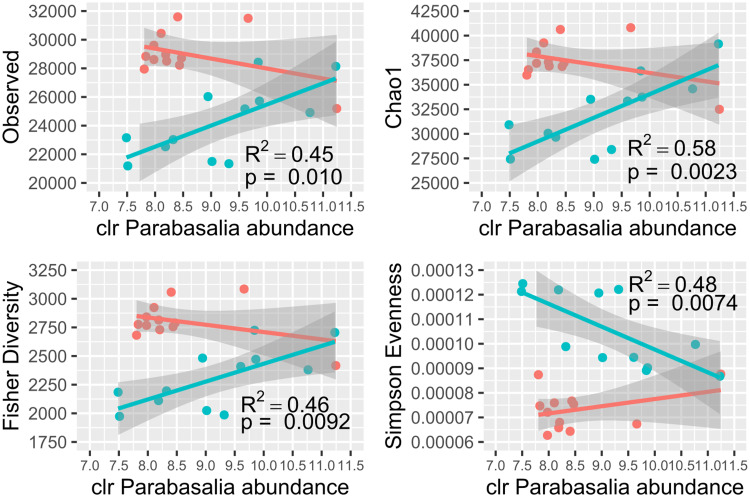
Relationship between Parabasalia abundance (normalized by centred log-ratio; clr) and microbial diversity metrics. Observed refers to the total number of observed taxa. Macaques with idiopathic chronic diarrhoea (ICD) and healthy controls are shown in pink and turquoise, respectively. Lines indicate separate linear regressions fitted for the ICD and control groups, and the shaded areas indicate 95% confidence intervals. The significant linear regression *P* values (<0.05) and corresponding R^2^ values derived from the ICD sample are indicated next to the corresponding line. The linear regression results for the control macaques were not significant (*P* value >0.05).

Our results suggested a possible relationship between parasite abundance and microbiota diversity. Amongst the macaques with ICD, there was a significant positive relationship between Parabasalia abundance and microbial alpha diversity measures (number of observed taxa, Chao1 and Fisher diversity). There was also a significant negative relationship between Parabasalia abundance and Simpson evenness, indicating a more non-uniform distribution of abundance amongst microbial taxa in animals with greater abundance of Parabasalia (Fig. 6). However, this may be restricted to the ICD condition, as a significant relationship between Parabasalia abundance and alpha diversity could not be demonstrated amongst the control macaques (*P* values derived from linear regression ranged from 0.21 to 0.27), although this is likely to have been influenced by the Parabasalia scarcity amongst the control animals (Fig. 5A). In addition, amongst the full cohort of macaques, using a combined linear model with disease state and Parabasalia abundance as predictors, only disease state showed a significant relationship with the same alpha diversity measures (*P* value derived from linear regression <0.0001), whereas Parabasalia abundance did not (*P* value ranged from 0.074 to 0.18). Intriguingly, ICD macaques with greater Parabasalia abundance appeared to more closely resemble control macaques in terms of alpha diversity.

**Fig. 7.**
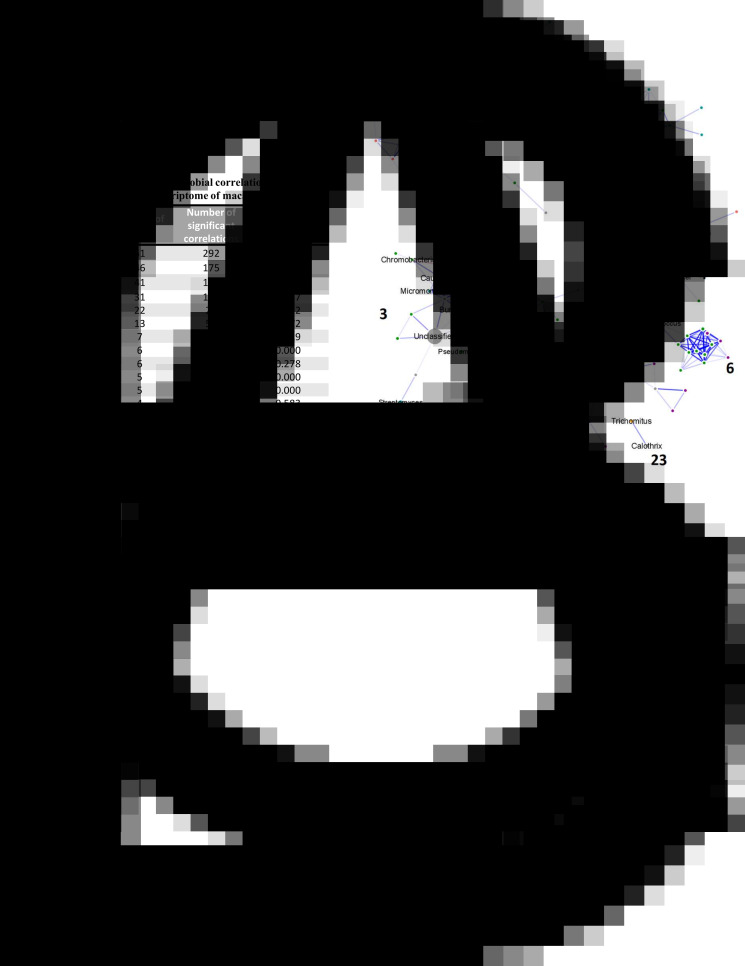
Correlation analysis of microbial abundance amongst macaques with idiopathic chronic diarrhoea. (A) DiVenn (Sun *et al*., 2019) figure showing overlap of positive (red) and negative (blue) correlations with bacteria amongst the parabasalid genera of interest. Yellow colour indicates shared correlations for which the direction of correlation differs between the groups. (B) Summary table for the microbial correlation networks. (C) Correlation network of the most abundant microbial genera (greater than 0.005% in at least 1 sample). Edges link nodes (genera) for which abundance was strongly correlated (sharing a significant correlation coefficient greater than 0.8). Node size is scaled to percentage abundance (ignoring the abundance of the ‘unclassified’ group), and labels for genera with less than 0.1% abundance are omitted (except for Parabasalia nodes and their immediate neighbours). Nodes are coloured according to phylum (Actinobacteria; turquoise, Bacteroides; pink, Firmicutes; purple, Proteobacteria; green and Parabasalia; orange) and edge transparency is scaled to the magnitude of the correlation coefficient. Edges linking to *Tetratrichomonas* are highlighted in red. Connected components are numbered sequentially by decreasing number of nodes and connected components with fewer than 7 nodes are not shown, except those that contain Parabasalia (21 connected components are not shown).

To investigate specific interactions between Parabasalia and bacterial members of the microbiota, we performed an all *vs* all correlation analysis at the genus level. We focused on the macaques with ICD and included only the most abundant taxa (greater than 0.005% in at least 1 sample). Amongst 358 taxa, with a total of 64 261 possible interactions, our results indicated 11 606 significant abundance correlations between genera. Of the 3 parabasalid genera of interest, *Tetratrichomonas* showed the greatest number of significant correlations with bacteria (110) followed by *Pentatrichomonas* (53), and *Trichomitus*, which showed far fewer significant correlations (17). *Tetratrichomonas* and *Pentatrichomonas* substantially overlapped in terms of bacterial genera showing significant positive and negative correlations, possibly indicating shared relationships with bacteria. In contrast, *Trichomitus* did not share common negative or positive relationships with any bacterial genera with either of the other parabasalid genera (Fig. 7A). Amongst the full complement of significant correlations, *Tetratrichomonas* stood out as participating in a large number of positive correlations. To investigate this further, we performed a network analysis by linking genera which shared a strong positive correlation. The genera were resolved into 29 connected components (Fig. 7). Of the 12 larger connected components (greater than 3 genera), 4 had a network clustering coefficient of greater than 0.5, indicating the majority of genera correlated with a given genus were also correlated with one another, suggesting well-supported and interdependent networks. Seventeen connected components had fewer than 4 genera, which in total accounted for 41 genera. This overall suggests a complex mixture of interdependent and independent bacterial genera. *Tetratrichomonas* inhabited the largest connected component of the 3 parabasalid genera of interest (network 3), with a moderate network clustering coefficient of 0.431, indicating a greatest potential interdependence with bacteria. Within network 3, the closeness centrality of *Tetratrichomonas* was 0.465, the 10th highest in the 41-node network, suggesting a relatively central hub-like position in comparison to most bacterial nodes. In contrast, *Pentatrichomonas* (network 7) and *Trichomitus* (network 23) inhabited smaller and more sparsely interconnected components, with 7 and 2 genera, respectively.

Notably, almost all bacterial genera which showed a significant negative correlation with *Tetratrichomonas* and *Pentatrichomonas* were Gram negative, but this pattern did not extend to *Trichomitus* (Table 2). *Pentatrichomonas* in particular showed a negative correlation with many bacterial genera reported to contain mucosal inhabitants which are opportunistic pathogens in various host species, including *Gemella* (Nazik *et al*., 2018), *Moraxella* (Goldstein *et al*., 2009), *Mannheimia* (Clawson and Murray, 2014) and *Aggregatibacter* (Karched *et al*., 2012). Importantly, *Tetratrichomonas* showed a strong negative correlation with *Prevotella*, which was the most abundant bacterial genus across all samples. The full list of significant correlations for *Tetratrichomonas*, *Pentatrichomonas* and *Trichomitus* amongst the macaques with ICD is shown in Table S4.

**Table 2.** Top significant negative correlations between parabasalid and bacterial genera by sparCC analysis

Parasite genus	Bacterial genus	Bacterial phylum	Bacterial Gram stain	Correlation coefficient	*P* value
*Tetratrichomonas*	*Leeuwenhoekiella*	Bacteroidetes	−	−0.824	0.00
	*Algibacter*	Bacteroidetes	−	−0.800	0.00
	*Desulfocapsa*	Proteobacteria	−	−0.793	0.00
	*Veillonella*	Firmicutes	−	−0.790	0.01
	*Desulfitobacterium*	Firmicutes	+	−0.784	0.00
	*Alteromonas*	Proteobacteria	−	−0.765	0.00
	*Emticicia*	Bacteroidetes	−	−0.751	0.00
	*Xenorhabdus*	Proteobacteria	−	−0.740	0.01
	*Prevotella*	Bacteroidetes	−	−0.721	0.00
	*Psychrobacter*	Proteobacteria	−	−0.718	0.00
*Pentatrichomonas*	*Basfia*	Proteobacteria	−	−0.735	0.00
	*Gemella*	Firmicutes	+/−	−0.675	0.00
	*Moraxella*	Proteobacteria	−	−0.639	0.03
	*Avibacterium*	Proteobacteria	−	−0.638	0.00
	*Acidaminococcus*	Firmicutes	−	−0.635	0.05
	*Roseburia*	Firmicutes	+	−0.630	0.03
	*Aggregatibacter*	Proteobacteria	−	−0.617	0.04
	*Mannheimia*	Proteobacteria	−	−0.614	0.03
	*Chania*	Proteobacteria	−	−0.614	0.04
	*Thalassospira*	Proteobacteria	−	−0.610	0.05
*Trichomitus*	*Dickeya*	Proteobacteria	−	−0.696	0.00
	*Kocuria*	Actinobacteria	+/−	−0.689	0.02
	*Amycolatopsis*	Actinobacteria	+	−0.633	0.05
	*Aerococcus*	Firmicutes	+	−0.614	0.03
	*Prochlorococcus*	Cyanobacteria	−	−0.545	0.04
	*Phascolarctobacterium*	Firmicutes	−	−0.527	0.05
	*Pseudonocardia*	Actinobacteria	+	−0.525	0.05
	*Desulfosporosinus*	Firmicutes	+	−0.508	0.05

The majority of relationships identified amongst the macaques with ICD were not consistent amongst the control group. Only 1188 out of 11 606 total significant relationships for the macaques with ICD were homodirectionally concordant amongst the control animals, including 2 out of 110 relationships for *Tetratrichomonas* (*Bradyrhizobium* and *Pentatrichomonas*), 5 out of 53 for *Pentatrichomonas* (*Acinetobacter*, *Janthinobacterium*, *Mesorhizobium*, *Streptomyces* and *Tetratrichomonas*) and 2 out of 17 for *Trichomitus* (*Calothrix* and *Colwellia*). This may indicate that the microbial community structure and interdependence was dramatically different between the ICD and control conditions.

We performed a differential abundance analysis between the ICD and control groups in order to investigate any potential impact of the parabasalids on disease aetiology. Interestingly, differential abundance analysis suggested a moderate significantly higher abundance of *Tetratrichomonas* and *Pentatrichomonas* (log_2_ fold differences were 1.62 and 1.80, respectively; adjusted *P* value <0.001), but not *Trichomitus*, amongst the macaques with ICD compared with the healthy controls. The original authors ruled out several known common GI pathogens (Bacteria: *Campylobacter jejuni*, *Salmonella*, *Shigella flexneri* and *Yersinia enterocolitica* and Parasites: *Cryptosporidium*, *Giardia*) as the cause of ICD by culture and microscopy-based methods. To complement this, we searched the dataset for potentially pathogenic viral lineage amongst the taxonomic profile. We focused on a selection of 29 potential primate-infecting eukaryotic viruses which we identified by the literature search (Oberste *et al*., 2007; Handley *et al*., 2012; Campanini *et al*., 2013; Janowski *et al*., 2017; Gao *et al*., 2018; Zhang *et al*., 2019*b*; Smura *et al*., 2020; Kang *et al*., 2021) (Fig. S7). Abundance of these viruses was low; total abundance of all 29 viruses was less than 1.6% for all animals, and the highest individual viral abundance was for Simian enterovirus 19, at 0.52%. Notably, we did not identify any significant difference in abundance for any of the viruses comparing between diseased and control animals (Mann–Whitney U test, *P* value >0.05).

In order to further query the potential influence of parabasalids on the microbiota, we examined the relationship between the HUMAnN2-annotated functional microbial gene expression and parabasalid abundance. The mean-variance relationship of the MetaCyc pathway quantification data is shown in Figure S8. A PCA of the ICD samples based on microbial pathway abundance showed tentative segregation of samples with low and high *Tetratrichomonas* abundance (Fig. S9). We identified a significant negative relationship between the abundances of 12 MetaCyc pathways and that of *Tetratrichomonas* amongst the macaques with ICD (Table 3), although the magnitude of the log_2_ fold changes were relatively small. The strongest relationship was detected for the Superpathway of *N*-acetylglucosamine, *N*-acetylmannosamine and *N*-acetylneuraminate degradation. The majority of functional sequences could not be attributed to a particular microbial species. However, many functions corresponded to likely constitutive bacterial functions such as peptidoglycan synthesis, potentially indicating a negative relationship with bacteria which could not be classified. The analysis did not identify any significant positive relationships between *Tetratrichomonas* and MetaCyc pathway abundances, and no significant relationships were found for the abundances of both *Pentatrichomonas* and *Trichomitus* amongst the macaques with ICD. The significant negative relationships identified for *Tetratrichomonas* could not be detected amongst the control animals.

**Table 3.** Microbial pathways showing a significant relationship with Tetratrichomonas abundance

MetaCyc ID	Description	Log_2_ fold change^a^	Average abundance (CPM)	Species stratification	Stratified abundance	Adjusted *P* value
GLCMANNANAUT-PWY	Superpathway of *N*-acetylglucosamine, *N*-acetylmannosamine and *N*-acetylneuraminate degradation	−1.28	5.19	NA		0.04
P441-PWY	Superpathway of *N*-acetylneuraminate degradation	−1.12	12.5	NA		0.03
PWY-6507	4-Deoxy-L-threo-hex-4-enopyranuronate degradation	−0.90	16.6	NA		0.03
PWY-7242	D-fructuronate degradation	−0.76	25.4	NA		0.04
GALACTUROCAT-PWY	D-galacturonate degradation I	−0.73	21.9	NA		0.04
GALACT-GLUCUROCAT-PWY	Superpathway of hexuronide and hexuronate degradation	−0.65	25.7	NA		0.04
GLUCUROCAT-PWY	Superpathway of *β*-D-glucuronosides degradation	−0.61	27.1	NA		0.04
PWY-5686	UMP biosynthesis	−0.44	38.7	*Treponema succinifaciens*	0.014	0.04
				*Bifidobacterium angulatum*	0.018	
				*Coprococcus catus*	0.050	
				*Eubacterium biforme*	0.061	
PWY-6386	UDP-*N*-acetylmuramoyl-pentapeptide biosynthesis II (lysine-containing)	−0.38	41.8	*Bifidobacterium angulatum*	0.0054	0.05
				*Treponema succinifaciens*	0.023	
				*Phascolarctobacterium succinatutens*	0.051	
				*Eubacterium biforme*	0.070	
PWY-6385	Peptidoglycan biosynthesis III (mycobacteria)	−0.37	46.9	*Treponema succinifaciens*	0.024	0.04
				*Phascolarctobacterium succinatutens*	0.130	
PWY-6387	UDP-*N*-acetylmuramoyl-pentapeptide biosynthesis I (meso-diaminopimelate containing)	−0.36	53.3	*Bifidobacterium angulatum*	0.0050	0.04
				*Treponema succinifaciens*	0.021	
				*Eubacterium biforme*	0.053	
				*Phascolarctobacterium succinatutens*	0.130	
PEPTIDOGLYCANSYN-PWY	Peptidoglycan biosynthesis I (meso-diaminopimelate containing)	−0.35	55.9	*Bifidobacterium angulatum*	0.0059	0.04
				*Treponema succinifaciens*	0.024	
				*Eubacterium biforme*	0.051	
				*Phascolarctobacterium succinatutens*	0.13	

a The coefficient resulting from the linear regression fit between pathway and *Tetratrichomonas* abundance, interpreted as the log_2_ fold change in pathway abundance per unit of *Tetratrichomonas* abundance.

## Discussion

We aimed to investigate potential relationships between the host, Trichomonads and the mucosal microbiota during health and disease through re-analysis of an existing metatranscriptomics dataset derived from macaque fecal samples. We identified a novel combination of *Pentatrichomonas*, *Tetratrichomonas* and *Trichomitus* parasites in the macaque gut. *P. hominis* was previously reported in laboratory macaques with ICD (Laing *et al*., 2018), and simian immunodeficiency virus (Zaragoza *et al*., 2011). However, to our knowledge, this is the first description of *Trichomitus* and *Tetratrichomonas* spp. colonizing the macaque gut. *Tetratrichomonas* spp. (Cepicka *et al*., 2006) and *Pentatrichomonas* spp. (Li *et al*., 2016; Bastos *et al*., 2018; Kim *et al*., 2020) are common GI-inhabitants of mammals. *Trichomitus* spp. typically infect reptiles and amphibians (Viscogliosi and Müller, 1998; Delgado-Viscogliosi *et al*., 2000), although mammalian infection has been reported (Dimasuay *et al*., 2013). Previous reports of macaque-infecting Trichomonads have also included gastric-localized *Tritrichomonas* spp. (Kondova *et al*., 2005). It is possible that the intestinal Trichomonad infections represent an artefact resulting from laboratory husbandry, as reports are scarce, and studies on wild macaques did not identify intestinal Trichomonads (Adhikari and Dhakal, 2018; Zhang *et al*., 2019*a*). As the animals were at times housed together (Westreich *et al*., 2019), the possibility of transmission of parasites between the laboratory animals seems likely. Any potential relationship between Trichomonads and diseases such as ICD in macaques has not been reported.

Our results suggested commonality in the expressed functional genes across the Trichomonads. We observed similar energy generation mechanisms for the macaque-infecting parabasalids as have been previously reported for *T. vaginalis*, demonstrated by a high abundance of transcripts associated with glycolysis, hydrogenosomal metabolism, amino acid catabolism (including the ADH pathway) and glycogen storage and processing (Müller, 1990; Kulda, 1999; Westrop *et al*., 2017). We also detected BspA expression potentially attributed to the macaque-infecting parabasalids. BspAs are of interest because proteins of this family have demonstrated roles in host adhesion by bacteria as well as adhesion between bacterial cells (Sharma, 2010). Importantly, *T. vaginalis* BspAs have been implicated in host adhesion *in vitro* (Handrich *et al*., 2019). In addition, *T. vaginalis* BspAs are differentially expressed in response to *Mycoplasma* symbionts, suggesting a potential role in parasite–bacteria interactions in modulating parasite binding to host cells (Margarita *et al*., 2022) and possibly binding to members of the microbiota too.

Our results also indicated a potential influential interaction between Trichomonads and microbial diversity in the macaque gut, as has been reported for other hosts and mucosa (El Sayed Zaki *et al*., 2010; Ji *et al*., 2020; Wei *et al*., 2020; Li *et al*., 2021). Probable coinfection with at least 3 Trichomonad genera complicated accurate abundance estimation due to the portion of sequences which could not be assigned to a specific genus (Watts *et al*., 2019). Thus, dissection of the relative effects for individual parasites was recalcitrant. This highlights a limitation of observational studies (Cani, 2018). Despite the greater abundance of *Trichomitus*, our results suggested *Tetratrichomonas* had the greatest abundance correlation with differences in the microbiota. *Tetratrichomonas* participated in the greatest number of significant abundance correlations, and was a central node within a densely interconnected microbial positive correlation network. Correlation networks have been effectively utilized to identify keystone species within microbial communities with biological significance (Duran-Pinedo *et al*., 2011). An overlap in specific relationships between differing Trichomonad spp. and bacteria may be suggested by shared correlations with bacterial abundance between *Pentatrichomonas* and *Tetratrichomonas*. In addition, of particular interest, a positive abundance correlation with *Prevotella*, which we observed for *Tetratrichomonas* in the macaque gut, has been described for *T. vaginalis* in the human UGT (Martin *et al*., 2013; Jarrett *et al*., 2019). Conserved interactions may result from biochemical features shared amongst the bacteria, supported by our observation that many of the bacteria negatively correlated with *Tetratrichomonas* and *Pentatrichomonas* were Gram negative. This is notable because other Trichomonads such as *Dientamoeba fragilis* (Chan *et al*., 1993) depend on Gram-negative bacteria for *in vitro* growth. Microbial interactions identified in this study varied hugely between the diseased and healthy conditions, similarly to previous results from the healthy and diseased human oral microbiota (Duran-Pinedo *et al*., 2011). This could suggest wholesale changes in community structure between conditions, but may also reflect unreliability in quantifying microbial abundance correlation (Weiss *et al*., 2016; Matchado *et al*., 2021). Although only a single sample, 1 control macaque both resembled the ICD macaques in terms of microbial profile, and showed the greatest abundance of Trichomonads, consistent with a potential parasite–bacterial interaction.

Previous studies have suggested the interaction between Trichomonads and the vaginal microbiota is bidirectional. The microbial profile can influence the ability of Trichomonads to colonize the mucosa (Rathod *et al*., 2011), and the presence of Trichomonad can perturb the microbial profile (Fichorova *et al*., 2013; Wei *et al*., 2020). However, the direction of influence between Trichomonads and the microbiota in the macaque gut could not be determined in the absence longitudinal data. In the macaque, Parabasalia expression of potential microbial-targeting genes such as lysozyme could indicate predation, as has been demonstrated for *T*. *vaginalis* (Pinheiro *et al*., 2018). This could provide a mechanistic basis for negative correlations between parasite and microbial abundance. However, we could only reliably attribute lysozyme-encoding transcripts to *Trichomitus*, whereas *Tetratrichomonas* was the only Trichomonad genus correlated with bacterial functional expression. Negative correlation of *Tetratrichomonas* abundance with bacterial degradation pathways for monosaccharides such as GlcNAc and Sia5NAc, potentially derived from mucin glycoproteins (Yurewicz *et al*., 1987) or microbial cells (Pinheiro *et al*., 2018), could indicate nutritional competition. This is supported by the detected expression of GlcNAc-targeting glycosyl hydrolases and potentially associated catabolic enzymes by the Trichomonads.

The absence of several known GI bacteria pathogens and microbial parasites was confirmed by culture and microscopy-based methods, and thus may be excluded as causative agents of ICD in the macaques. We performed an additional search for potentially pathogenic viruses amongst the datasets. However, we did not identify any clear differences for any putative host-infecting virus when comparing between diseased and control animals, suggesting viral infection may not be the primary cause of ICD. A greater abundance of reads classified as originating from the *Campylobacter* genus amongst the animals with ICD was originally reported (Westreich *et al*., 2019), and so the potential presence of other pathogens in this genus cannot be ruled out. Our results did not establish a causal link between Trichomonads and ICD in macaques. The higher abundance of *Pentatrichomonas* and *Tetratrichomonas* could indicate a causal role in disease. High *P. hominis* abundance in macaques with ICD was previously reported, but not causally liked to disease (Laing *et al*., 2018). However, abundance of these parasites appeared to promote a more diverse (control-like) microbiota. Trichomonads were positively correlated with microbial diversity, which was also higher in healthy animals, and has been considered characteristic of healthy human gut (Malard *et al*., 2021). This contrasts with previous work which suggested that the presence of *T. gallinae* and *Tritrichomonas musculis* decreases GI microbial diversity (Ji *et al*., 2020; Wei *et al*., 2020). Notably, we did not detect any correlation between Trichomonads and the abundance of bacterial genes underlying mucin degradation or fucose utilization, the previously proposed determinants of macaque ICD (Westreich *et al*., 2019). It is feasible that the ICD state provides a beneficial environment for Trichomonad colonization, within which the parasites exert a disruptive influence. This is supported by the observation that Trichomonad–microbial interactions appeared to be highly dependent on disease state.

Our results revealed a relatively low parasite abundance in the macaque fecal samples, highlighting the need for greater sequencing depth or selective target enrichment (Gaudin and Desnues, 2018) to quantitatively study the parasite transcriptome *in vivo*. Reference sequences from closely related parasite strains would also have greatly facilitated analysis (Breitwieser *et al*., 2019). As is typical for a diverse *in vivo* metatranscriptome (Li *et al*., 2019), a large proportion of sequences could not be assigned to a specific phylum.

In summary, these metatranscriptomics analyses of Trichomonads in the macaque gut have provided the first *in vivo* insight into Trichomonad mucosal colonization, which validates numerous *in vitro* studies (Müller, 1990; Kulda, 1999; Westrop *et al*., 2017; Handrich *et al*., 2019). Our findings support previous reports of Trichomonad–microbiota interactions (Ji *et al*., 2020; Wei *et al*., 2020; Bierlein *et al*., 2021), and demonstrate that such interactions vary between parasite species and are highly context-dependent. Longitudinal studies, or those involving experimental Trichomonad infection, could be used to investigate causality and underlying mechanisms in the parasite–microbiota–disease interrelationship.

## Data Availability

This work presents a re-analysis of metatranscriptomics data generated by Westreich *et al*. (2019), DOI: https://doi.org/10.1186/s40168-019-0664-z. Original data are available from the NCBI SRA database (Leinonen *et al*., 2011) under accession numbers SRX3517701-SRX3517724. All the supplementary files containing *de novo* assemblies of the original sequence data and alignments used for phylogenetics are available *via* figshare (https://figshare.com/s/5d6f50cb71ed2ffc82fb).

## Supplementary material

For supplementary material accompanying this paper visit https://doi.org/10.1017/S0031182022001688. click here to view supplementary material
