# Supplementary material for: Revisiting fecal metatranscriptomics analyses of macaques with idiopathic chronic diarrhoea with a focus on trichomonad parasites
Source: Parasitology. 2022 Dec 12;150(3):248–61. doi: 10.1017/S0031182022001688 (PMC10090643; doi:10.1017/S0031182022001688)
Supplement: Supplementary file 1 [file S0031182022001688sup.zip › S0031182022001688sup001.docx]

## Supplementary Tables

Table S1. Summary statistics for analysis of macaque faecal metatranscriptomics data.

| **Sample Name** | **Disease Status** | **SRA Accession (Westreich, et al. 2019)** | **Total reads** | **Percentage classified (Kraken2)** | **Total Parabasalia reads (Kraken2)** | **Percentage reads aligned to assembly*** | **Assembly N50 (bp)** |
| --- | --- | --- | --- | --- | --- | --- | --- |
| Macaque 1 | ICD | SRR6425383 | 93378446 | 44.05 | 41658 | 92.18 | 975 |
| Macaque 2 | ICD | SRR6425419 | 91765624 | 44.36 | 979543 | 88.93 | 556 |
| Macaque 3 | ICD | SRR6425425 | 84565457 | 43.31 | 149035 | 90.20 | 633 |
| Macaque 4 | ICD | SRR6425431 | 94858184 | 45.33 | 253473 | 92.70 | 1021 |
| Macaque 5 | ICD | SRR6425437 | 84358150 | 40.85 | 331386 | 91.68 | 787 |
| Macaque 6 | ICD | SRR6425443 | 91324252 | 48.40 | 140086 | 91.00 | 738 |
| Macaque 7 | ICD | SRR6425449 | 95389943 | 43.36 | 319351 | 90.86 | 681 |
| Macaque 8 | ICD | SRR6425455 | 85807851 | 40.71 | 260884 | 91.21 | 743 |
| Macaque 9 | ICD | SRR6425461 | 81029034 | 42.96 | 80196 | 91.22 | 796 |
| Macaque 10 | ICD | SRR6425323 | 90191208 | 45.20 | 35177 | 91.75 | 917 |
| Macaque 11 | ICD | SRR6425329 | 93708838 | 45.29 | 75020 | 92.74 | 916 |
| Macaque 12 | ICD | SRR6425335 | 86445134 | 43.61 | 692880 | 91.07 | 667 |
| Macaque 13 | Control | SRR6425341 | 88679444 | 37.25 | 47900 | 91.11 | 647 |
| Macaque 14 | Control | SRR6425347 | 79805026 | 38.60 | 72956 | 89.91 | 618 |
| Macaque 15 | Control | SRR6425353 | 85607222 | 38.57 | 73397 | 90.71 | 661 |
| Macaque 16 | Control | SRR6425359 | 93086319 | 38.15 | 40010 | 90.60 | 659 |
| Macaque 17 | Control | SRR6425365 | 83904127 | 40.06 | 1338929 | 90.62 | 674 |
| Macaque 18 | Control | SRR6425371 | 100470693 | 39.61 | 51563 | 90.05 | 680 |
| Macaque 19 | Control | SRR6425377 | 92829650 | 42.33 | 40718 | 90.45 | 686 |
| Macaque 20 | Control | SRR6425389 | 97193278 | 36.87 | 66225 | 90.08 | 568 |
| Macaque 21 | Control | SRR6425395 | 86705938 | 38.03 | 225302 | 89.54 | 582 |
| Macaque 22 | Control | SRR6425401 | 96725446 | 36.53 | 58957 | 91.83 | 655 |
| Macaque 23 | Control | SRR6425407 | 98867658 | 33.23 | 46448 | 92.07 | 605 |
| Macaque 24 | Control | SRR6425413 | 85554278 | 34.48 | 55489 | 90.62 | 544 |

*Proportion of total reads which were uniquely aligned to the metatranscriptome assembly using STAR.

**Table S2.** List of annotated trichomonad contigs encoding 18S rRNA, actin and EF-1α – see separate excel file. Corresponding sequences are in Data file S1 to be accessed via Figshare: <https://figshare.com/s/5d6f50cb71ed2ffc82fb>

**Table S3** – see separate excel file**.** List of annotated contigs encoding candidate protein from Parabasalia (using *T. vaginalis* genes). Corresponding sequences are in Data file S2, to be accessed via Figshare: <https://figshare.com/s/5d6f50cb71ed2ffc82fb>

**Table S4** – see separate excel file**.** Full list of significant Bacterial genera correlated with the presence of *Tetratrichomonas*, *Pentatrichomonas* and *Trichomitus* amongst the macaques with ICD.

Supplementary figures

**
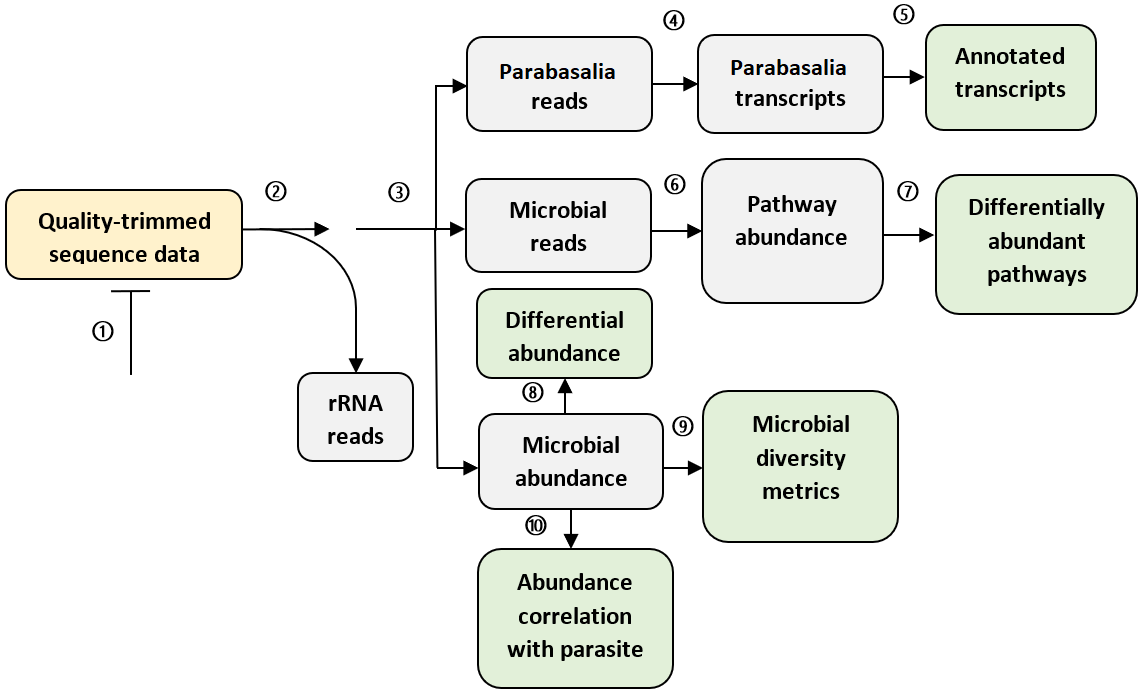
**

**Figure S1.** Flow chart describing the workflow used to quantitatively analyse macaque metatranscriptomics data. 1. Read quality assessment with FastQC (Andrews 2018), 2. Alignment to a prokaryotic and eukaryotic rRNA database with SortMeRNA (Kopylova, et al. 2012), 3. k-mer search of the NCBI non-redundant nucleotide database (O'Leary, et al. 2016) with Kraken2 (Wood, et al. 2019), 4. *de novo* assembly with rnaSPAdes (Bushmanova, et al. 2019), 5. Transcript alignment to the reference with BLASTx (Altschul, et al. 1990), 6. Functional classification with HUMAnN2 (Franzosa, et al. 2018), 7. Differential abundance test by the Limma-trend method (Law, et al. 2014) using the R package edgeR (Robinson, et al. 2010), 8. Microbial differential abundance test by ANCOM-BC (Lin and Peddada 2020), 9. Microbiota profiling with R packages PhyloSeq (McMurdie and Holmes 2013) and Microbiome (Lahti and Shetty 2017), 10. Microbial correlation analysis using sparCC (Friedman and Alm 2012).

| **A 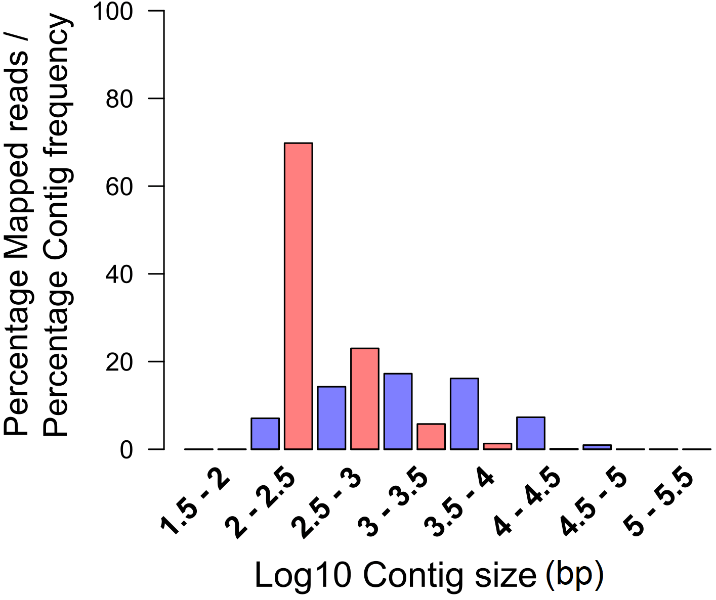** | **B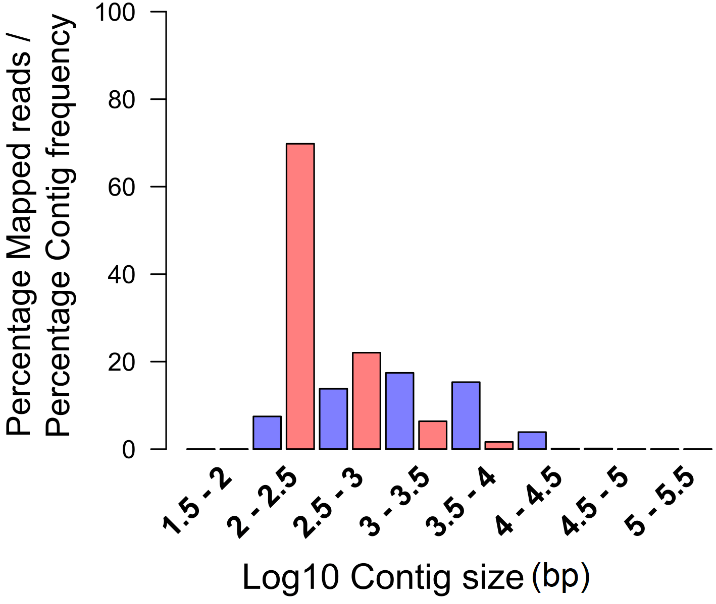** |
| --- | --- |

Figure S2. Quality control statistics for de novo assembly of the macaque faecal metatranscriptome. (A) control macaques and (B) macaques with ICD. Red bars show a frequency histogram for contig length amongst the assembly, and blue bars indicate the proportion of reads which aligned to contigs of each size range. Values are presented as a percentage of the total number of reads or contigs.


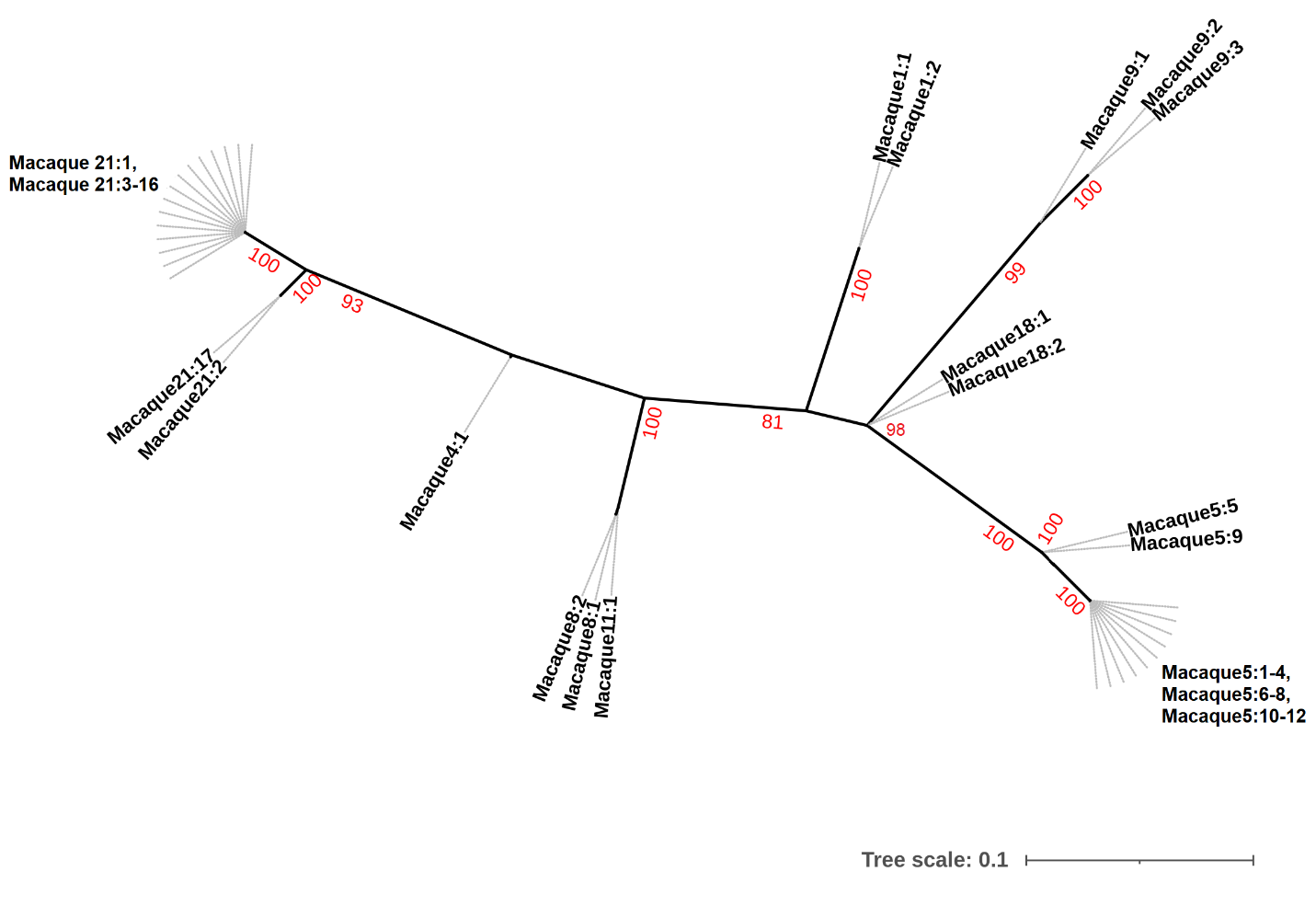


Figure S3. Unrooted maximum likelihood phylogeny (K2P model allowing unequal transition and transversion rates and equal base frequencies, with gamma rate heterogeneity) of Parabasalia-like 18S rRNA sequences in the macaque faecal metatranscriptome. Bootstrap values (1000 replicates) greater than 75% are shown on branches in red. Units for tree scale are inferred substitutions per base pair. Grey dashed lines link leaf labels to the tips of branches, but are not included in the branch length. Sequences are named sequentially according to the animal from which they originated, e.g. Macaque1:1, Macaque1:2.


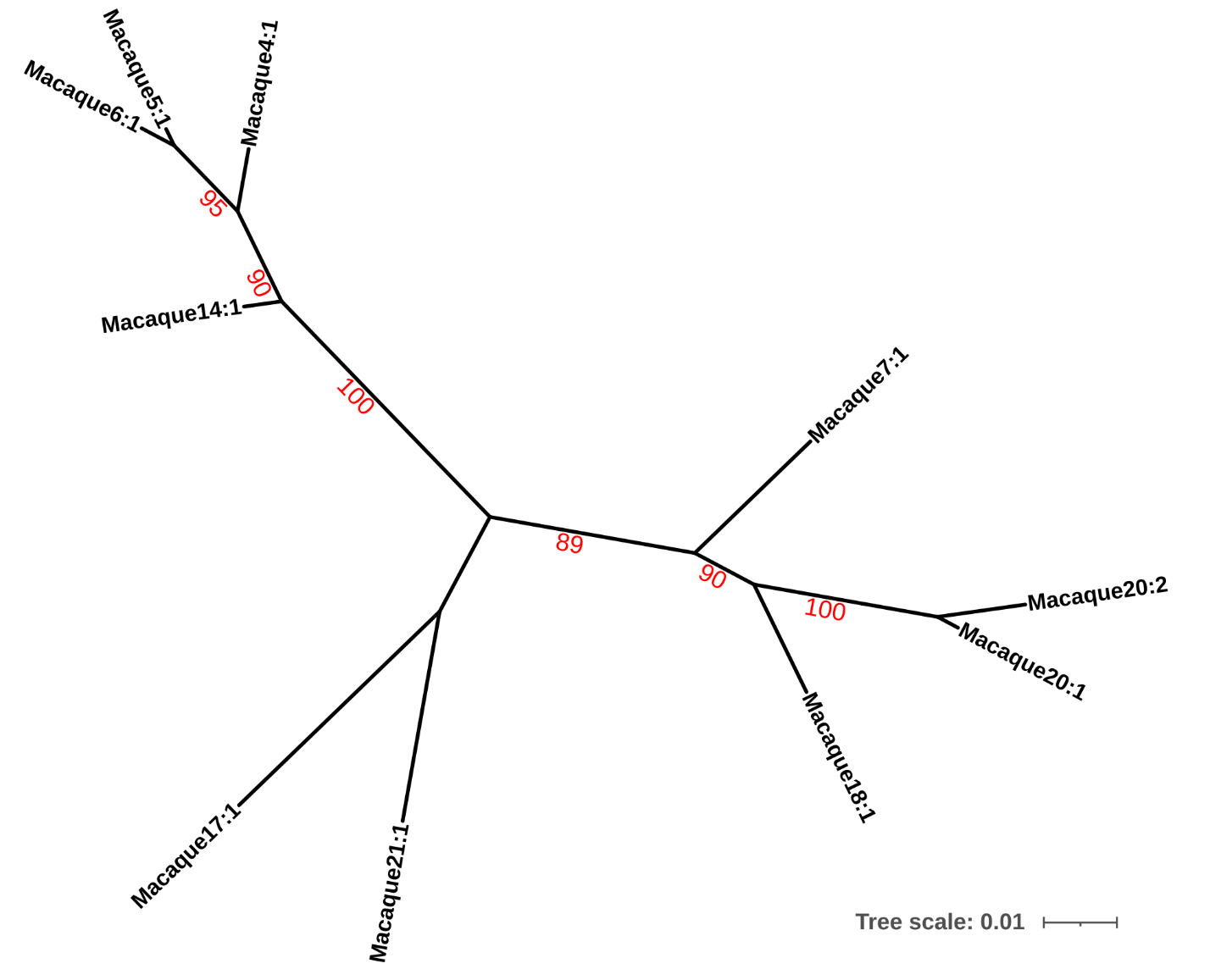


Figure S4. Unrooted maximum likelihood phylogeny (TVM transversion model allowing unequal base frequencies, with empirical base frequencies and invariable sites) of Parabasalia-like actin sequences in the macaque faecal metatranscriptome. Bootstrap values (1000 replicates) greater than 75% are shown on branches in red. Units for tree scale are inferred substitutions per base pair. Sequences are named sequentially according to the animal from which they originated, e.g. Macaque1:1, Macaque1:2.


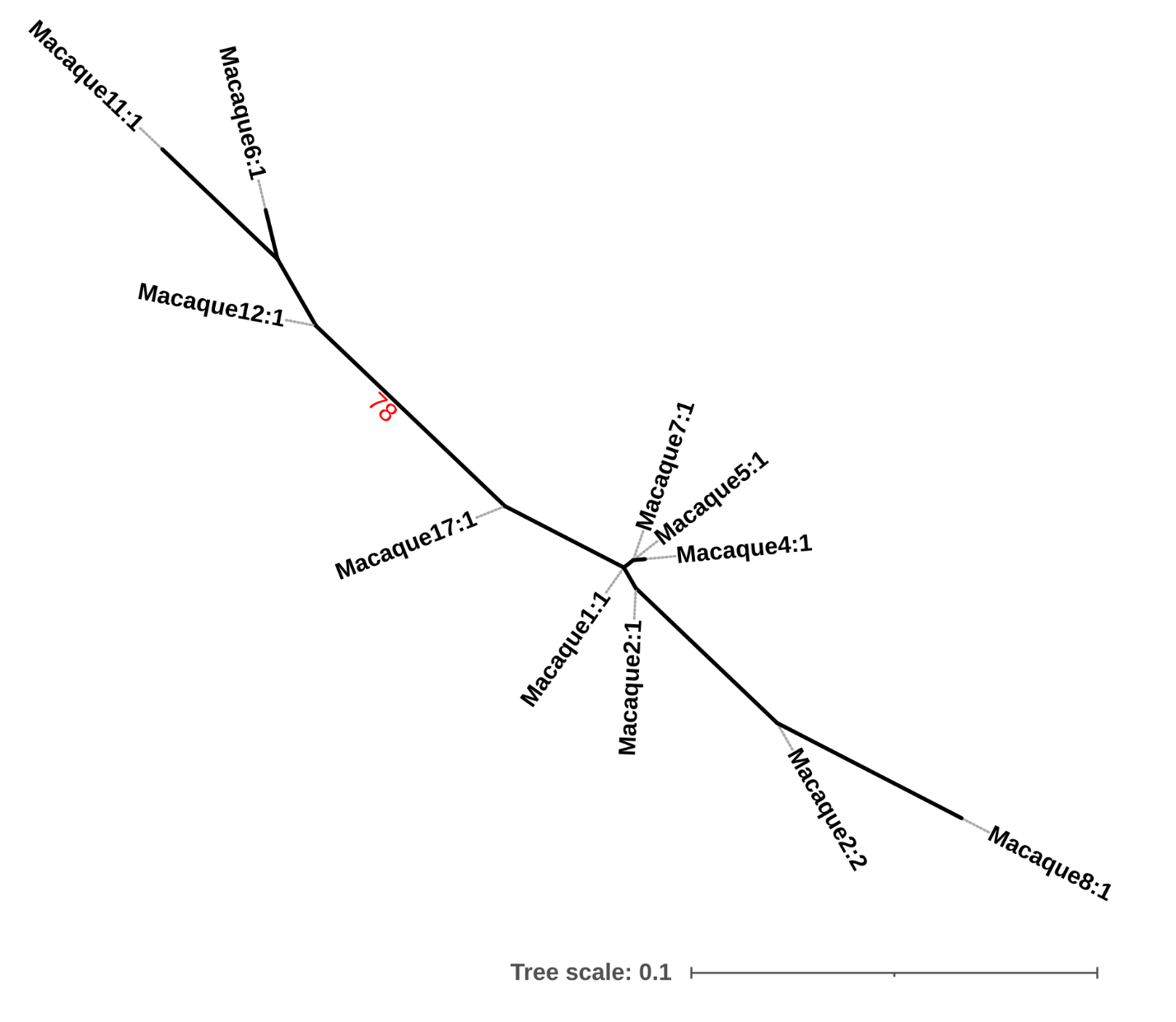


Figure S5. Unrooted maximum likelihood phylogeny (TN model allowing unequal transition and transversion rates, unequal base frequencies and unequal purine and pyrimidine rates, with empirical base frequencies and invariable sites) of Parabasalia-like EF-1α sequences in the macaque faecal metatranscriptome. Bootstrap values (1000 replicates) greater than 75% are shown on branches in red. Units for tree scale are inferred substitutions per base pair. Grey dashed lines link leaf labels to the tips of branches, but are not included in the branch length. Sequences are named sequentially according to the animal from which they originated, e.g. Macaque1:1, Macaque1:2.

**
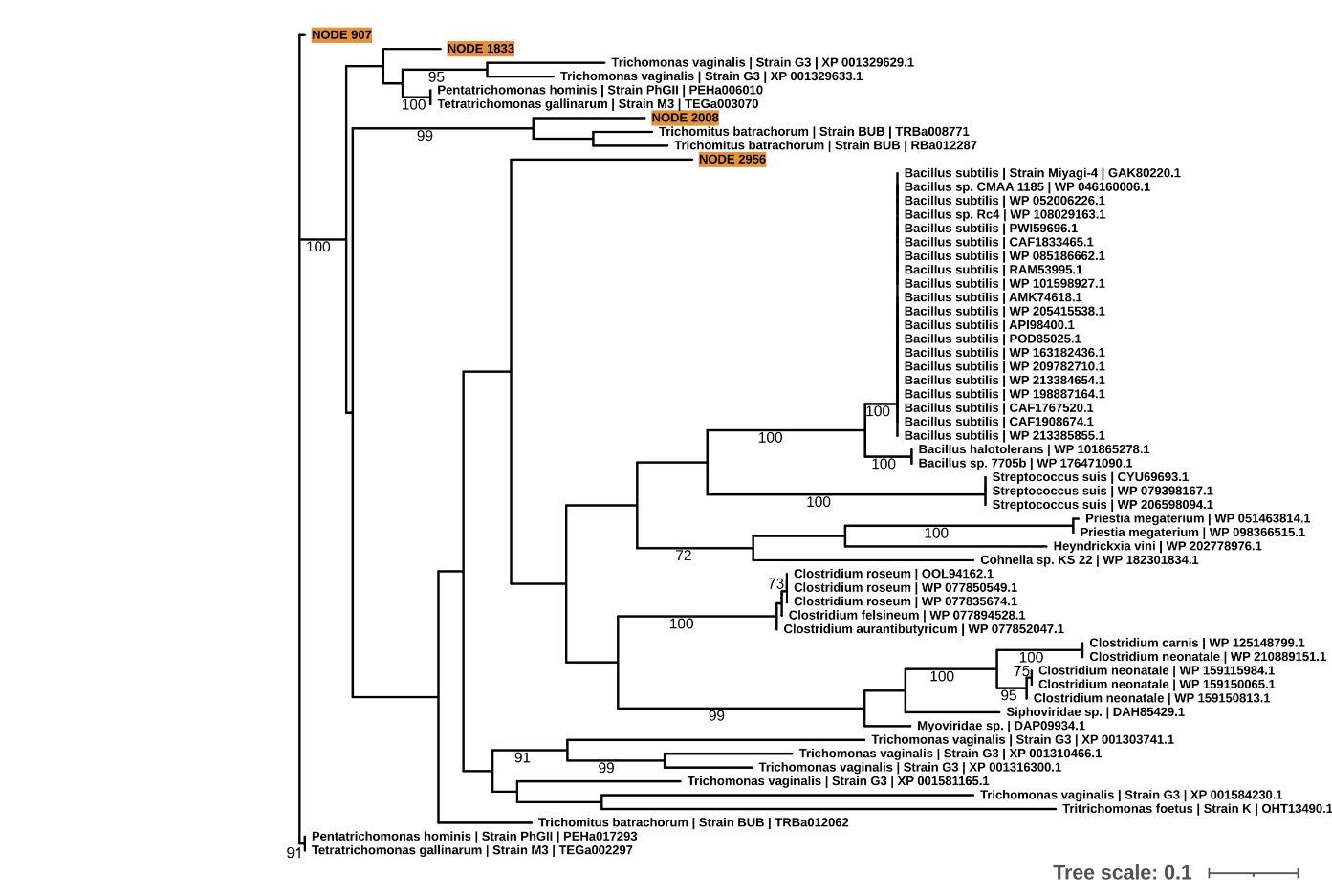
**

Figure S6. Unrooted maximum likelihood phylogeny (LG model with gamma rate heterogeneity) of Parabasalia-like lysozyme sequences in the macaque faecal metatranscriptome (highlighted in orange) alongside a range of homologous sequences. Bootstrap values (1000 replicates) greater than 70% are shown on branches. Units for tree scale are inferred substitutions per amino acid residue. Where available, Genbank accessions (Benson, et al. 2015) are shown at the end of tip labels.

**
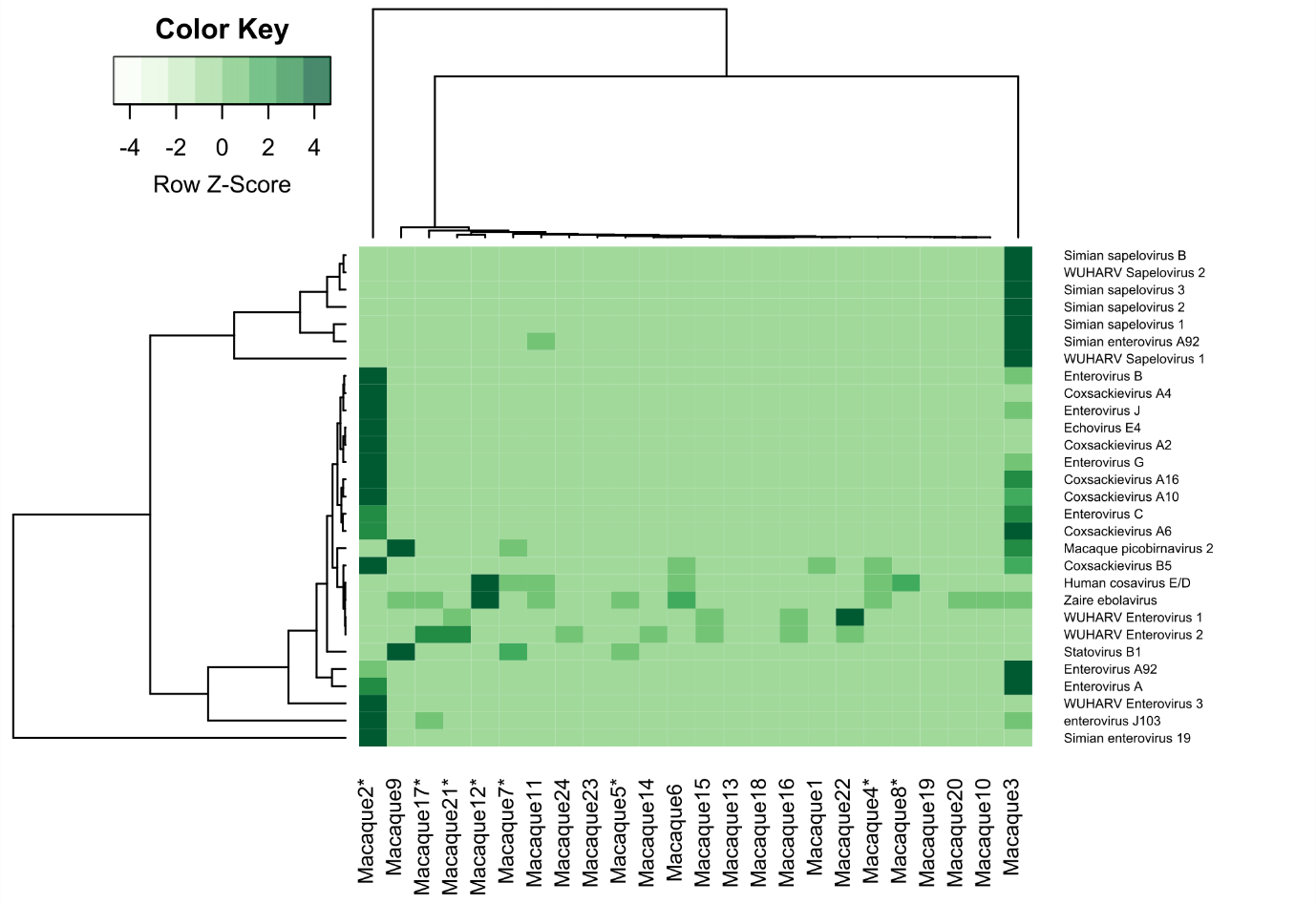
**

**Figure S7.** Abundance heatmap for viral sequences derived from faecal samples from macaques with ICD (Macaque 1-12) and healthy controls (Macaque 13-24). Only viruses identified from the literature as putatively infecting the macaque host are included search (Oberste, et al. 2007; Handley, et al. 2012; Campanini, et al. 2013; Janowski, et al. 2017; Gao, et al. 2018; Zhang, et al. 2019; Smura, et al. 2020; Kang, et al. 2021). Abundance was normalised as a fraction of library size and normalised by the Z-score method. Animals with a total abundance of Parabasalia greater than 0.25% are marked with an asterisk.


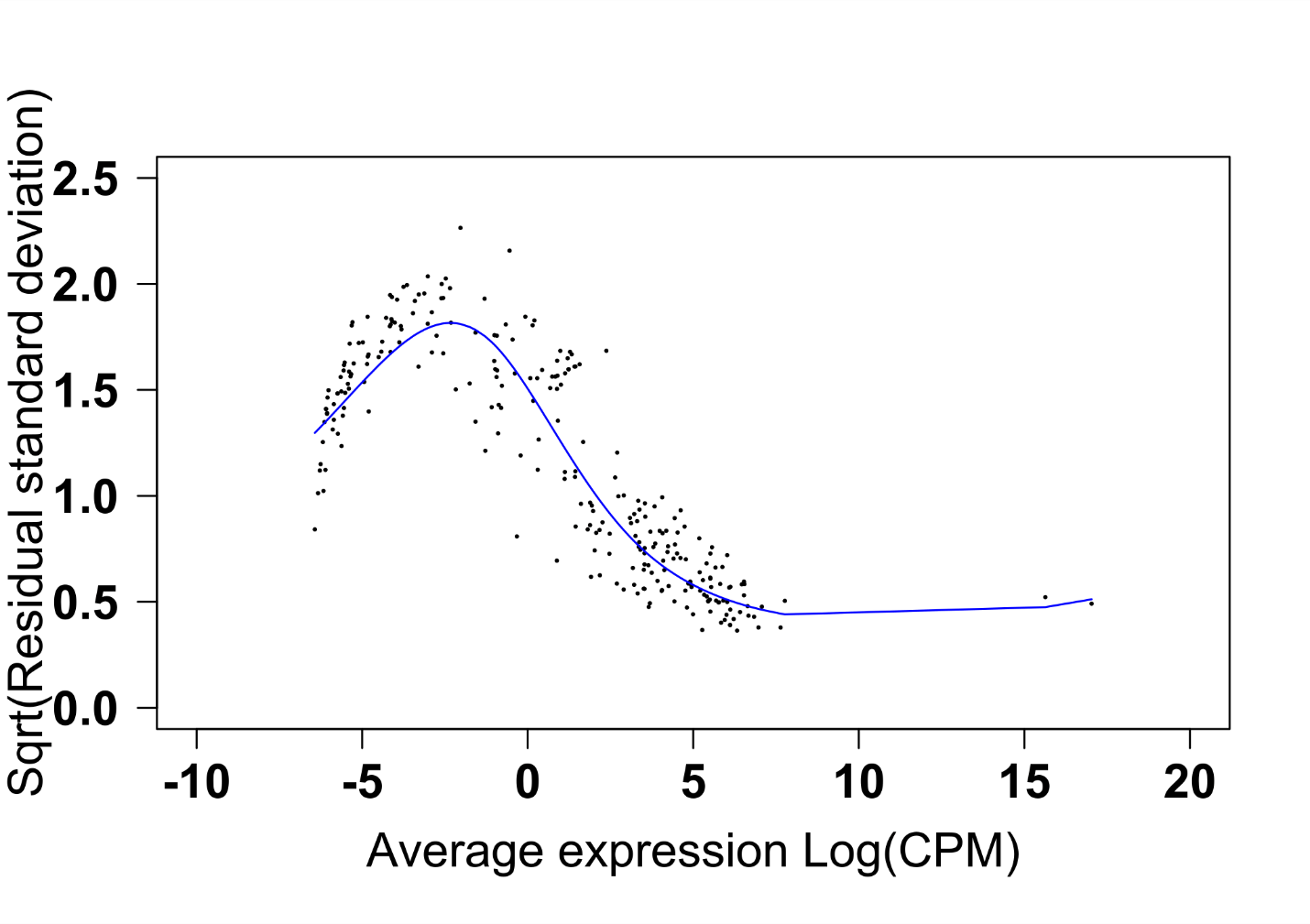


Figure S8. Mean-variance relationship for microbial MetaCyc pathway abundance amongst macaques with ICD.


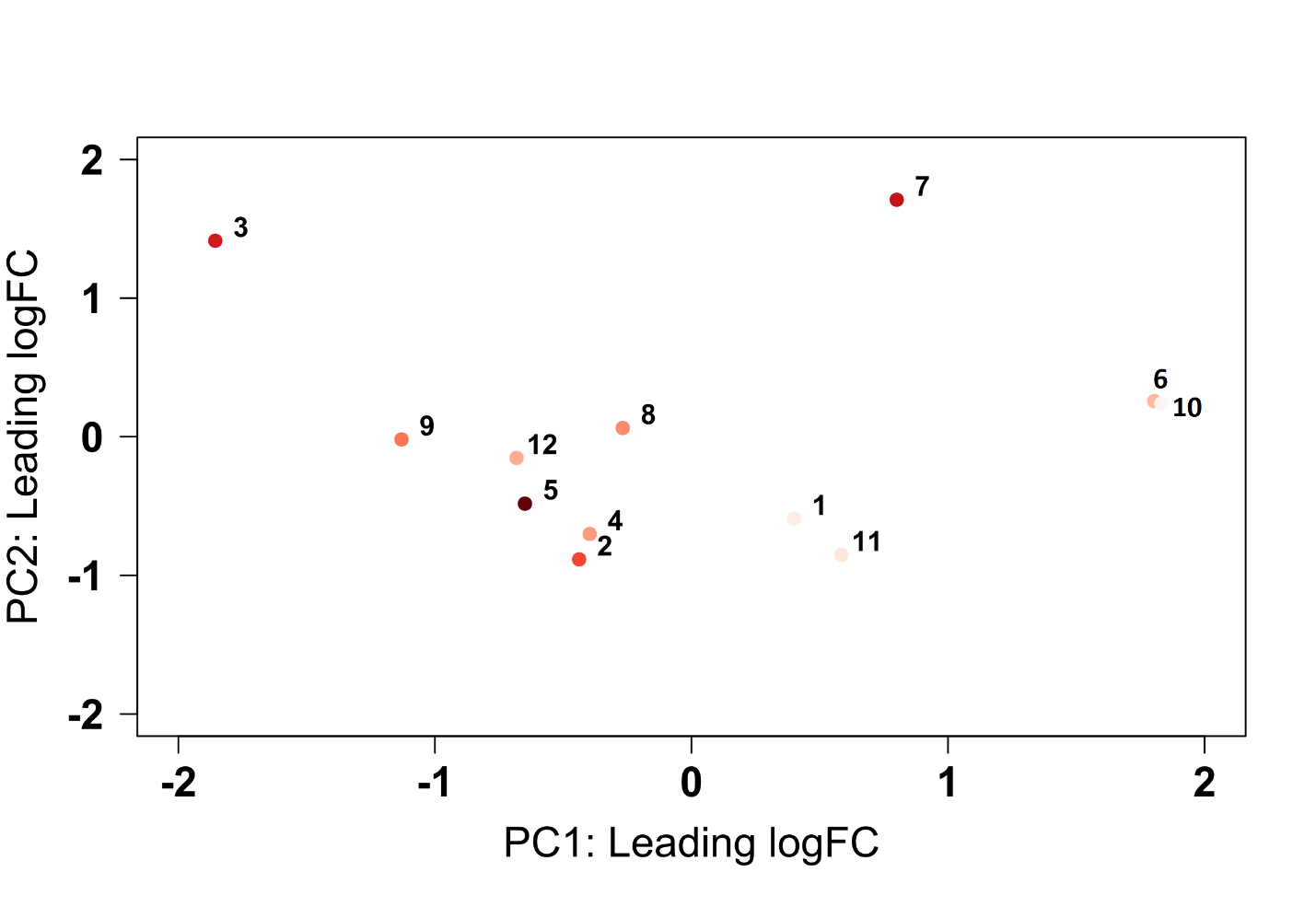


**Figure S9.** PCA analysis of ICD macaque metatranscriptome samples based on the abundances of microbial MetaCyc pathways. Points are shaded according to the clr normalised abundance values for *Tetratrichomonas*.

**Cited references**

Altschul SF, Gish W, Miller W, Myers EW, Lipman DJ. 1990. Basic local alignment search tool. *Journal of Molecular Biology* 215:403-410.

FastQC [Internet]. Babraham bioinformatics; 2018 [cited 2018 29/4/18].

Benson DA, Clark K, Karsch-Mizrachi I, Lipman DJ, Ostell J, Sayers EW. 2015. GenBank. *Nucleic Acids Research* 43:D30-D35.

Bushmanova E, Antipov D, Lapidus A, Prjibelski AD. 2019. rnaSPAdes: a de novo transcriptome assembler and its application to RNA-Seq data. *GigaScience* 8.

Campanini G, Rovida F, Meloni F, Cascina A, Ciccocioppo R, Piralla A, Baldanti F. 2013. Persistent human cosavirus infection in lung transplant recipient, Italy. *Emerg Infect Dis* 19:1667-1669.

Franzosa EA, McIver LJ, Rahnavard G, Thompson LR, Schirmer M, Weingart G, Lipson KS, Knight R, Caporaso JG, Segata N, et al. 2018. Species-level functional profiling of metagenomes and metatranscriptomes. *Nature Methods* 15:962-968.

Friedman J, Alm EJ. 2012. Inferring correlation networks from genomic survey data. *PLOS Computational Biology* 8:e1002687.

Gao F, Bian L, Hao X, Hu Y, Yao X, Sun S, Chen P, Yang C, Du R, Li J, et al. 2018. Seroepidemiology of coxsackievirus B5 in infants and children in Jiangsu province, China. *Hum Vaccin Immunother* 14:74-80.

Handley SA, Thackray LB, Zhao G, Presti R, Miller AD, Droit L, Abbink P, Maxfield LF, Kambal A, Duan E, et al. 2012. Pathogenic simian immunodeficiency virus infection is associated with expansion of the enteric virome. *Cell* 151:253-266.

Janowski AB, Krishnamurthy SR, Lim ES, Zhao G, Brenchley JM, Barouch DH, Thakwalakwa C, Manary MJ, Holtz LR, Wang D. 2017. Statoviruses, A novel taxon of RNA viruses present in the gastrointestinal tracts of diverse mammals. *Virology* 504:36-44.

Kang HJ, Yoon Y, Lee YP, Kim HJ, Lee DY, Lee JW, Hyeon JY, Yoo JS, Lee S, Kang C, et al. 2021. A Different Epidemiology of Enterovirus A and Enterovirus B Co-circulating in Korea, 2012-2019. *J Pediatric Infect Dis Soc* 10:398-407.

Kopylova E, Noé L, Touzet H. 2012. SortMeRNA: fast and accurate filtering of ribosomal RNAs in metatranscriptomic data. *Bioinformatics* 28:3211-3217.

Lahti L, Shetty S. 2017. Tools for microbiome analysis in R. In.

Law CW, Chen Y, Shi W, Smyth GK. 2014. Voom: Precision weights unlock linear model analysis tools for RNA-seq read counts. *Genome Biology* 15:R29.

Lin H, Peddada SD. 2020. Analysis of compositions of microbiomes with bias correction. *Nature Communications* 11:3514.

McMurdie PJ, Holmes S. 2013. phyloseq: an R package for reproducible interactive analysis and graphics of microbiome census data. *PLoS One* 8:e61217.

O'Leary NA, Wright MW, Brister JR, Ciufo S, Haddad D, McVeigh R, Rajput B, Robbertse B, Smith-White B, Ako-Adjei D, et al. 2016. Reference sequence (RefSeq) database at NCBI: current status, taxonomic expansion, and functional annotation. *Nucleic Acids Research* 44:D733-745.

Oberste MS, Maher K, Pallansch MA. 2007. Complete genome sequences for nine simian enteroviruses. *J Gen Virol* 88:3360-3372.

Robinson MD, McCarthy DJ, Smyth GK. 2010. edgeR: a Bioconductor package for differential expression analysis of digital gene expression data. *Bioinformatics* 26:139-140.

Smura T, Blomqvist S, Kolehmainen P, Schuffenecker I, Lina B, Böttcher S, Diedrich S, Löve A, Brytting M, Hauzenberger E, et al. 2020. Aseptic meningitis outbreak associated with echovirus 4 in Northern Europe in 2013-2014. *J Clin Virol* 129:104535.

Westreich ST, Ardeshir A, Alkan Z, Kable ME, Korf I, Lemay DG. 2019. Fecal metatranscriptomics of macaques with idiopathic chronic diarrhea reveals altered mucin degradation and fucose utilization. *Microbiome* 7:41.

Wood DE, Lu J, Langmead B. 2019. Improved metagenomic analysis with Kraken 2. *Genome Biology* 20:257.

Zhang W, Kataoka M, Doan HY, Ami Y, Suzaki Y, Takeda N, Muramatsu M, Li TC. 2019. Characterization of a Novel Simian Sapelovirus Isolated from a Cynomolgus Monkey using PLC/PRF/5 Cells. *Sci Rep* 9:20221.
